# Formation of Lanthanide Luminescent Aggregates With RNA Using Tripodal Europium [Eu(III)] BTA Self‐Assembled Complexes

**DOI:** 10.1002/chem.71601

**Published:** 2026-08-25

**Authors:** Tómas A. Gudmundsson, Changzhuang Bai, Grace E. Maynard, Pall Thordarson, Thorfinnur Gunnlaugsson

**Affiliations:** ^1^ School of Chemistry and Trinity Biomedical Sciences Institute Trinity College Dublin The University of Dublin College Green Dublin Ireland; ^2^ Advanced Materials and BioEngineering Research (AMBER) Centre Trinity College Dublin The University of Dublin Dublin Ireland; ^3^ School of Chemistry and the UNSW RNA Institute The University of New South Wales Sydney NSW Australia

**Keywords:** bioimaging, coordination, lanthanide luminescence, RNA Aggregation, supramolecular assembly, terpy

## Abstract

A terpyridine‐appended β‐peptide and its tripodal benzene‐1,3,5‐tricarboxamide (BTA) derivative formed luminescent Eu(III) self‐assembled materials, showing significant enhanced metal ion centered emission upon torula yeast RNA binding in buffered pH 7 HEPES solution due to the formation of luminescent aggregates as probed by using imaging platforms; attributed to changes in the water coordination environment of the Eu(III) center upon binding to the phosphate di‐ester backbone of the RNA.

## Graphical abstract

The two Eu(III) complexes of **1.Eu** and **2.Eu**, that were formed in situ, were used to bind to yeast RNA and **A10** (RNA) in a buffered pH 7 solution. The interaction gave rise to changes in the lanthanide‐centered emission because of direct binding of these complexes, which resulted in aggregation of the RNA, as demonstrated using phase‐contrast and atomic force microscopy.

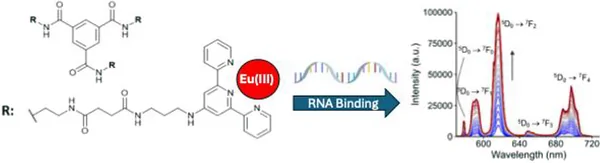

## 1 Introduction

There exists a significant interest in the development of luminescent systems that can selectively “*switch on”* their emission upon recognition or targeting of specific biomarkers, or through changes in their local environments [1, 2, 3, 4, 5, 6, 7, 8, 9, 10]. Exploiting supramolecular interactions has facilitated developments in chemical biology [11, 12, 13], where the design of photoactive agents for bioimaging can provide vital information about localized phenomena to identify diseases [14, 15, 16, 17, 18, 19]. Of particular interest is the capability to target nucleic acids such as RNA to facilitate the understanding of biological pathways [20, 21, 22, 23, 24, 25, 26], inspect cellular damage [27, 28], and even to detect strands from SARS‐CoV‐2 [29, 30].

A powerful strategy to design probes for RNA detection is through designing complexes based on lanthanide ions such as Eu(III), as they retain the small size and biocompatibility of organic molecules, while preserving their unique beneficial photophysical properties [14, 15, 16, 31], without resorting to the complex synthesis typically required for fluorescent labeling experiments [14, 15, 16, 19, 32, 33, 34]. Examples include systems where the emission properties are exploited for RNA‐responsive dual‐targeted bioimaging [35], and further applications by the surface functionalization of AuNPs [22] for luminescent sensing^23^], biosensing [24], or imaging microdamage in bone structures [25]. In the past, we developed cyclen‐based lanthanide complexes that promote phosphodiester hydrolysis of HPNP and RNA oligonucleotides [36, 37].

The Gunnlaugsson group has previously studied tripodal BTA‐based Eu(III) complexes as luminescent materials and for use in crosslinking of self‐assembled materials [38, 39]. We foresaw that by incorporating Eu(III) onto higher symmetry platforms, such as the *C_3_*‐symmetric benzene‐1,3,5‐tricarboxamide (BTA, or trimesic acid) unit, their potential to bind to RNA could be increased significantly, where the high coordination requirements of Eu(III) can be capitalized on, such as through interactions at the phosphodiester backbone [40]. Water‐soluble derivatives of the BTA platform have recently attracted interest in biological systems and have already shown promise in studying cell/material interactions [41, 42, 43, 44]. The outcome of our investigation is detailed below.

### 1.1 Design and Synthesis of Ligands **1** and **2**

In the work presented herein, ligands **1** and **2** were employed as potential candidates for forming aggregates with RNA in solution Figure 1. Ligand **1** resembled an unnatural *β*‐peptide capped with a terpyridine (*tpy*) moiety to act as an antenna upon coordination with Eu(III), while **2** is the tripodal derivative of **1** bound to the BTA platform, which may have increased potential to bind RNA.

**FIGURE 1:**
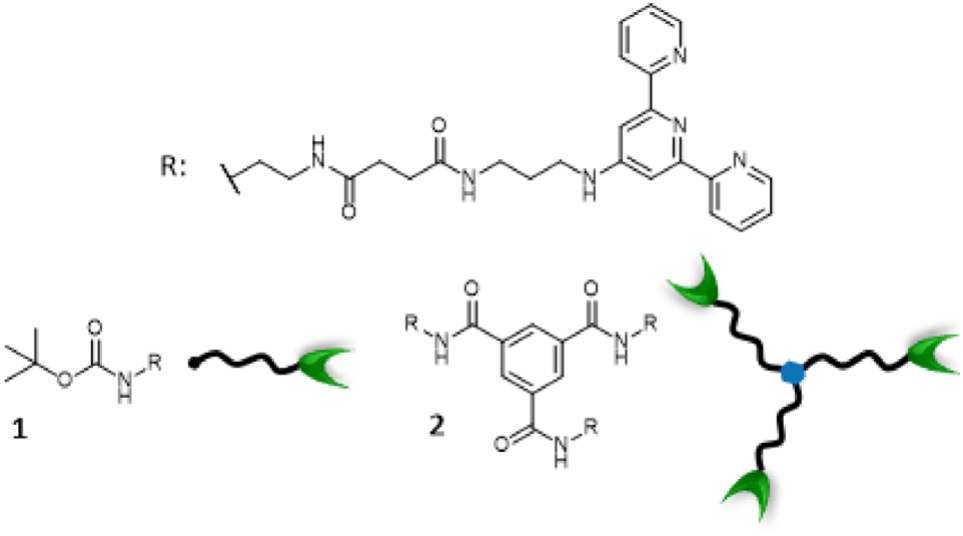
Chemical structures of **1** and **2** developed in this work, and the cartoon representation. The ligands were then used for the formation of the Eu(III) complexes **1.Eu** and **2.Eu**.

The synthesis and characterization of ligands **1** and **2** were performed in a few steps (See Schemes S1 and S2, respectively), both involving the use of the **tpy** terminal compound **3**. The formation of **3** involved refluxing the commercially available 4‐chloro‐tpy in neat 1,3‐diaminopropane at 120°C. The high reaction temperature facilitated nucleophilic aromatic substitution, while the vast molar excess of 1,3‐diaminopropane meant that any di‐substituted side products were avoided. The reaction was left to run for 18 h, and the resultant solution was precipitated out in water, giving the desired product **3** in 98% yield. In the case of compound **1**, the synthesis of the rest of the arm began by reacting BOC protection of ethylene‐1,2‐diamine to give **4** in 83% yield, which was then reacted in toluene with a minor excess of succinic anhydride at 60°C, over 18 h, the desired product **5** being isolated upon precipitation and washing with toluene, giving **5** in a 78% yield. Following this, **3** was coupled to **5** by using a standard peptide coupling reaction using EDCI·HCl, in dry DCM along with a stoichiometric amount of triethylamine and HOBT, at room temperature for 4 days. After a series of aqueous acid/base washes, compound **1** was successfully isolated in moderate yield of 52% (See Supporting Information for full characterization of **1**).

The synthesis of **2**, which we have recently communicated [45], where **2** was used in the formation of soft materials in a range of organic and alcoholic solvents, which could be crosslinked using both *d‐* and *f‐*metal ions, including Eu(III). However, the use of **2** in aqueous solution had not been previously investigated. As for **1**, synthesis of **2** involved the use of **3**, which was coupled to the BTA ligand **8** (See Scheme S1) using a peptide coupling method like that described above.

### 1.2 In Situ Formation of **1.Eu** and **2.Eu** and Interactions With RNA

The objective of this work was to employ ligands **1** and **2** in aqueous buffered solution to recognize and bind to RNA and induce supramolecular aggregation. In our previous work using **2**, we showed that in a 1:1 EtOH:MeCN solvent mixture, Eu(III) bound to the *tpy* moiety, with concomitant changes in the ground and the excited state of **2**, with the emerging of the Eu(III) emission at long wavelength due to sensitization of the ^5^D_0_→^7^F_J_ (J = 0–4) excited states of Eu(III), through an energy transfer from the S_1_ state via T_1_ upon excitation of the *tpy* moiety of ligand **2** 45]. Unfortunately, both ligands **1** and **2** showed poor solubility in either water or buffered solutions on their own. Therefore, they were initially dissolved in a minimum amount of DMSO through the aid of sonication, followed by heating with various equivalents of Eu(III) in HEPES buffer (10 mM) at pH 7.0 (See Figure S1), to form the Eu(III) complexes of these two ligands. The HEPES buffer was chosen to avoid significant interactions of the buffer with Eu(III) [46], and a pH of 7.0 was selected to avoid the often‐observed hydrolysis [36, 37] of RNA by hydrated Eu(III) complexes, particularly under slightly alkaline conditions [47, 48]. This method allowed for the formation of both **1.Eu** and **2.Eu**, which were found to be stable in solution, which consisted of 99% HEPES and 1% DMSO at pH 7 (See Figure S2 for the absorption and the emission spectra **1.Eu** and **2.Eu**).

The complexes employed in the aggregation studies herein were formed by using 1:1 stoichiometric ratio of Eu(III) to *tpy*; that is, 1 eq. of Eu(III) to **1** and **3** eq. of Eu(III) to **2**. For all experiments, the molarity of **2** solutions was then kept at a third of that of **1,** which enabled us to make a direct comparison between the two species upon interacting with RNA. However, as mentioned above, the interactions/binding of **2** with Eu(III) had only been probed in organic or in a mixture of alcohol:organic solvents such as EtOH:CH_3_CN mixtures [45]. Furthermore, the spectroscopic properties of **1** had not been investigated at all, and therefore, it was important to probe the ground and the excited state properties of both ligands in the more competitive 99% HEPES: 1% DMSO solution upon titration with Eu(III).

### 1.3 Spectroscopic Investigation of **1.Eu** and **2.Eu** in pH 7 HEPES Buffered Solutions

The ligands **1** and **2** (1.0 × 10^−5^ M) were titrated with stock solutions of Eu(III) (using EuCl_3_) from 0 → 3.0 equivalents to probe the changes in the ground and the singlet excited state properties of the *tpy* unit. Concomitantly, the delayed Eu(III) centered emission was monitored (by observing the Eu(III) phosphorescence emission). Analysis of these changes allowed us to determine the stoichiometry, the stability constants, and the speciation distribution for the resulting complexes formed in situ (under kinetic control). Hence, the absorbance, fluorescence emission and excitation, and the phosphorescent emission spectra were all recorded upon titration with Eu(III), and the changes in these spectra of **2** are presented in Figure 2 (the changes for **1** are given in Figure S3).

**FIGURE 2:**
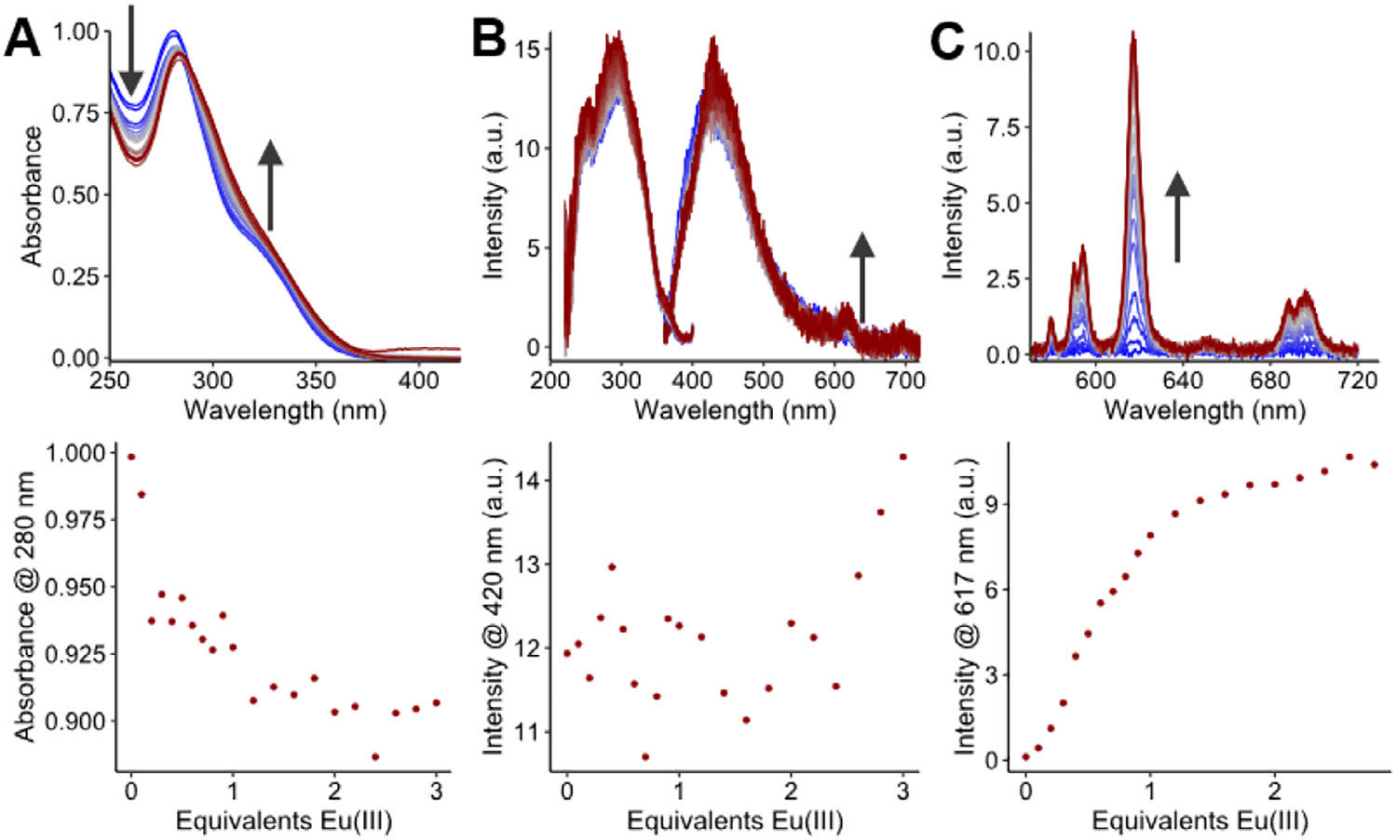
The spectroscopic results obtained for the titration of **2** (1.0 × 10^−5^ M) with 0 → 3.0 equivalents EuCl_3_ in HEPES (10 mM) pH 7.0 buffered solution (1% DMSO) where the full spectra and binding isotherms are presented for: A) Absorbance, B) Fluorescence emission (*λem* = 320 nm) and excitation (*λem* = 420 nm), and C) Phosphorescence emission (*λex* = 320 nm).

As can be observed from Figure 2 A,B, the changes in the absorption (with a small 2 nm red shift was seen for the tpy π−π* transition band at 280 nm) and the fluorescence emission spectra of the *tpy* units were overall minor; the latter being slightly enhanced upon addition of Eu(III). In contrast, the emerging Eu(III) emission (recorded upon excitation at 330 nm) showed all the ^5^D_0_ → ^7^F*_J_* (*J* = 1‐4) transitions emerging upon binding of Eu(III) to the *tpy* units. Notable is the presence of the electronic‐dipole *J* = 0 transition, which indicates a high symmetry within the complex and that the Laporte selection rules are not relaxed. These changes were analyzed using nonlinear regression analysis (ReactLab) [49]. Modelling ML species was successful and returned the stability constant for **1.Eu** β_1:1_ = 4.0 ± 0.9 and for **2.Eu** β_1:1_ = 5.0 ± 0.9 (See Figures S4 and S5). These binding constants appeared low (when compared to those reported for **2.Eu** in organic solvents) [45], however, this was likely a consequence of the high polarity of the solvent decreasing the stability of the complexes [50]. From these binding values, the speciation distribution was evaluated, and at 3.0 equivalents of Eu(III), approximately 25% of **1** had converted to the ML species, while ca. 75% of **2** had been formed. It is worth pointing out that the ML species values have significant error associated with them, which translates into ML with possible as high as ca. ± 25% and ± 34% error associated with these two complexes.

By measuring the excited state decay in the presence of 3 eq. of Eu(III) at the 617 nm Eu(III) transition, the formation of a species, which was best fitted to a single‐exponential decay, for each complex was observed (Supporting Information the decay profiles with associated fits: Figure S6 and Table 1). The determination of the lifetimes of each complex in both H_2_O and D_2_O solutions allowed us to determine the hydration number *q* (the number of metal bound water molecules) of each species in solution (See Equation S1) [51]. The lifetimes of **2.Eu** were significantly longer than **1.Eu**. The results are shown in Table 1, demonstrating that **2.Eu** had a q‐value of 2, while **1.Eu** had a much higher value of 6, which also explained the shorter excited state lifetimes of this system.

**TABLE 1:** Results from quantum yield experiments, lifetimes, and hydration numbers for **1.Eu** and **2.Eu** in the presence and absence of RNA in buffered H_2_O and D_2_O solutions based on 99:1 ratio discussed above.

	RNA	*Φ* (%)	*τ*_H2O_ (ms)	*τ*_D2O_ (ms)	*q* (± 0.1)
**1.Eu**	−	0.1	0.110	0.289	6.0
**1.Eu**	+	0.3	0.559	2.216	1.1
**2.Eu**	−	0.6	0.352	1.242	2.0
**2.Eu**	+	2.4	0.680	2.213	0.9

### 1.4 Interactions **1.Eu** and **2.Eu** and With RNA

Having established the formation of **1.Eu** and **2.Eu** in the buffered solution, titrations were undertaken with incremental addition of torula yeast RNA into **1.Eu** and **2.Eu** solutions at pH 7. The results for the changes in the Eu(III) emission **2.Eu** are shown in Figure 3A,B, demonstrating an increase in the Eu(III)‐centred emission for all the transitions ^5^D_0_→^7^F_J_ (J = 0–4). Such changes were also seen for **1.Eu** (See Figure S7), the changes in the Δ*J* = 2 transition, which corresponds to the hypersensitive transition centered at 617 nm, being enhanced 50‐fold for **1.Eu** and 10‐fold for **2.Eu,** Figure 3C. Despite the lower relative Eu‐centered enhancement, the emission of **2.Eu** was found to be four times greater than that of **1.Eu** at maximum due to the greater emission in the absence of RNA. The limit of detection (LOD) was calculated from the changes in the yeast RNA titration, using the signal‐to‐noise ratio (See Equation S2). The LOD for **1.Eu** was calculated as 91 ± 6 ng/mL, while for **2.Eu** it was determined as 89 ± 3 ng/mL at a 95% confidence level (i.e., no significant difference was observed) [52, 53, 54].

**FIGURE 3:**
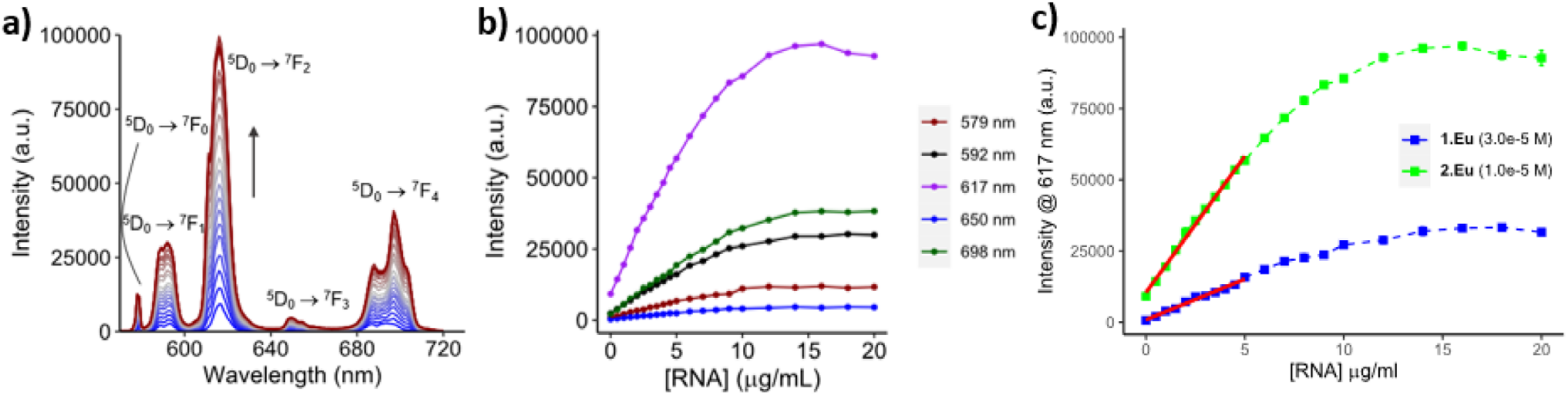
(a) Full phosphorescence emission spectra from the addition of yeast RNA to **2.Eu** (3.0 × 10^−5^ M) at pH 7.0 in aqueous HEPES (10 mM, pH 7.0)/DMSO (99:1, v/v) buffer. (b) The corresponding changes in the various Δ*J* transitions **2.Eu**. (c) The emission at 617 nm from **1.Eu** (1.0 × 10^−5^ M) and **2.Eu** (3.0 × 10^−5^ M) with calibration lines through each linear response region (right). See Supporting Information for full RNA titrations of **1.Eu**.

To investigate whether these complexes could interact with other RNA systems, these titrations were repeated using purified homo‐adenine decamer of RNA (**A10**) in place of torula yeast RNA. These titrations gave similar results to the heterogeneous yeast RNA, as demonstrated in Figure 4, for **2.Eu** (both titrations shown for comparison) as well as the titration isotherm as a function of yeast RNA both complexes.

**FIGURE 4:**
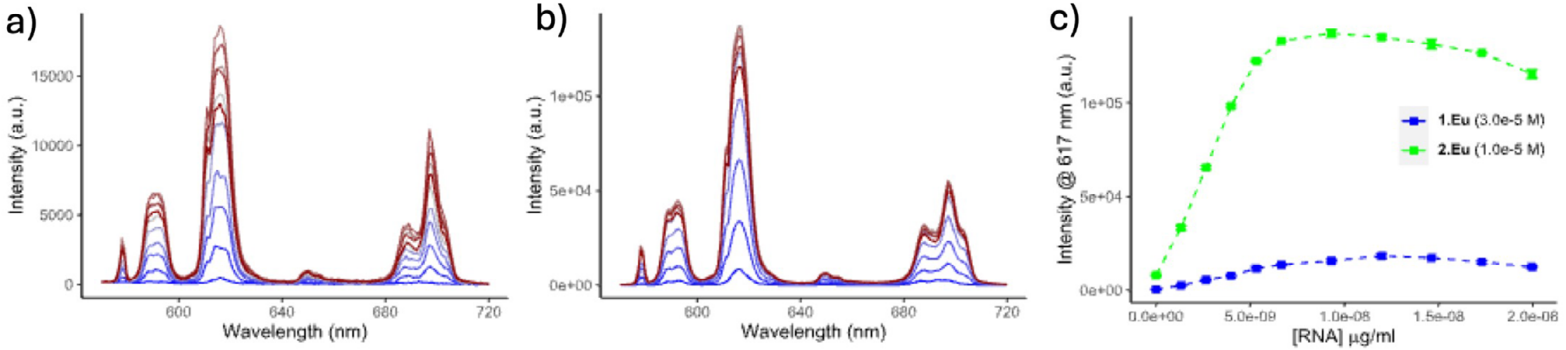
Eu(III) emission spectral changes from the titration of A10 into solutions of: (a) **1.Eu** (3.0 × 10^−5^ M) and (b) **2.Eu** (1.0 × 10^−5^ M) in aqueous HEPES (10 mM, pH 7.0)/DMSO (99:1, v/v) buffer. (c) Emission tracked at 617 nm of both solutions upon addition of A10.

Quantum yield experiments were also performed for both complexes before and after binding with torula yeast RNA. Before adding RNA, the quantum yield of **1.Eu** was very low at 0.1% and for **2.Eu** the quantum yield was determined as 0.3% Table 1. Once yeast RNA was mixed with the complexes, the quantum yields increased to 0.6% for **1.Eu**, with a more significant 2.4% enhancement for **2.Eu** (confirming the formation of more emissive species). While these values are low, they are within normally seen for lanthanide complexes in such competitive media, particularly where the hydration number *q*‐values are larger than unity. Therefore, *q* was also determined again for the complexes upon binding to yeast RNA (See Figure S8). Indeed, these measurements demonstrated that displacement of these metals bound water molecules upon interaction of the complexes with RNA, as when “saturated” with RNA, the hydration of **1.Eu** decreased from *q* ≈ 6 to *q* ≈ 1, while for **2.Eu** the decrease was from *q* ≈ 2 to *q* ≈ 1. This further helps in explain the higher background emission of **2.Eu** and the higher relative emission enhancement of **1.Eu**, seen and discussed above, as more metal‐bound water molecules were displaced upon RNA binding. Additionally, the binding of **2.Eu** to RNA would be more entropically favorable, and stronger overall binding could be expected, with greater emission enhancement then for **1.Eu**.

### 1.5 Hill Plot Analysis **1.Eu** and **2.Eu** With RNA

Titrations were also carried out where the **1.Eu** and **2.Eu** complexes were titrated into a fixed concentration of the yeast RNA. Plotting the changes in the Eu(III) emission as a function of the complex concentration (from 0 → 3.0 × 10^−5^ M and 0→1.0 × 10^−5^ M for the **1.Eu** and **2.Eu**, respectively). Gave a *s*igmoidal binding curve for both systems (See Figure S9). The Hill model is particularly useful as only the mass of the yeast RNA was known, where many other models require knowledge of the molarity. The resulting curves were fitted to the Hill equation to yield the Hill coefficient for cooperativity *n* = 4.3 ± 0.5 for **1.Eu** and *n* = 3.7 ± 0.2 for **2.Eu** revealing a highly cooperative self‐assembly mechanism but no significant difference between complexes (See Equation S3). Unsurprisingly, the model could not provide reliable *K_d_* values as the underlying equilibria here are likely to be a complex mixture of **1.Eu** “direct” interactions with RNA forming discrete complexes versus self‐association of those discrete complexes into higher‐order complexes in a cooperative rather than isodesmic fashion (as suggested by the Hill plot).

### 1.6 Investigating the Binding of **1.Eu** and **2.Eu** With RNA Using Microplate Reader Assay and Phase‐contrast Microscopy

To assess the binding of the **1.Eu** and **2.Eu** complexes to RNA further, a microplate reader assay was performed where the absorbance of various concentrations of **1.Eu**, **2.Eu** and Eu(III) mixed with a fixed concentration of 1 mg/mL torula yeast RNA was measured (See Figure S10). A scattering (turbidity) profile was observed going beyond 700 nm for the **1.Eu** and **2.Eu** solutions revealed that aggregation occurred upon interaction with RNA Figure 5, (left), while no significant scattering was observed for the solution containing only Eu(III) (See Figure S11). Scattering increased with a higher complex concentration until **2.Eu** reached a plateau, while a minor decrease was observed for **1.Eu**. Mixing **1.Eu** or **2.Eu** with RNA (1:1 mass ratio) showed increased scattering indicative of self‐assembly or aggregation formation, even at low concentrations.

**FIGURE 5:**
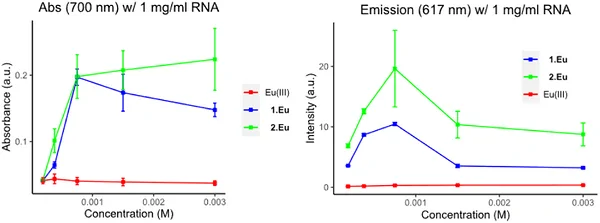
Absorbance (scattering) at 700 nm for various concentrations of **1.Eu**, **2.Eu** and Eu(III) mixed with 1 mg/mL of yeast RNA (left) in aqueous HEPES (10 mM, pH 7.0)/DMSO (99:1, v/v) buffer and emission of the same solutions at 617 nm.

The Eu(III) emission was also evaluated using a microplate reader assay where the Eu(III) luminescence of each solution was compared before and after adding RNA in a plate reader assay (See Figure S12). Here, enhancement in the characteristic delayed lanthanide emission from Eu(III) for **1.Eu** and **2.Eu** (being absent for Eu(III) alone) upon RNA binding was observed, which piqued our interest to investigate further the interactions between RNA and **1.Eu** and **2.Eu**, with the view of correlating how the tripodal structure could affect the binding and the resulting photophysical response that could then be correlated to aggregation or not using microscopic imaging.

Prior to that, and to account for any ligand aggregation, the excitation spectrum for ligand **1** (3.0 × 10^−5^ M) in the buffer solution was recorded and shown to be in good agreement with the absorption spectrum of **1**, which was not the case for **2** at 1.0 × 10^−5^ M (See Figure S1). However, upon addition of Eu(III), to give 1:1 and 3:1 ratio with **1** and **2**, respectively, and hence form **1.Eu** and **2.Eu** in situ, both solutions showed good agreement between these spectra, indicating no aggregation in solution upon formation of the complexes (See Figure S2).

Moreover, at high concentrations, the Eu(III) luminescence of **1.Eu** and **2.Eu** decreased due to scattering and nonradiative quenching. These samples were inspected further by using phase‐contrast microscopy, which showed aggregation at high concentrations Figure 6. Some aggregation was observed when mixing only Eu(III) with RNA, which was not reflected by scattering in the plate reader assay. Further, AFM imaging of dried RNA solutions showed simple aggregates that increased in size when mixed with **1.Eu** or **2.Eu** (See Figure S13), which indicated a successful interaction, or binding of **1.Eu** and **2.Eu** with RNA.

**FIGURE 6:**
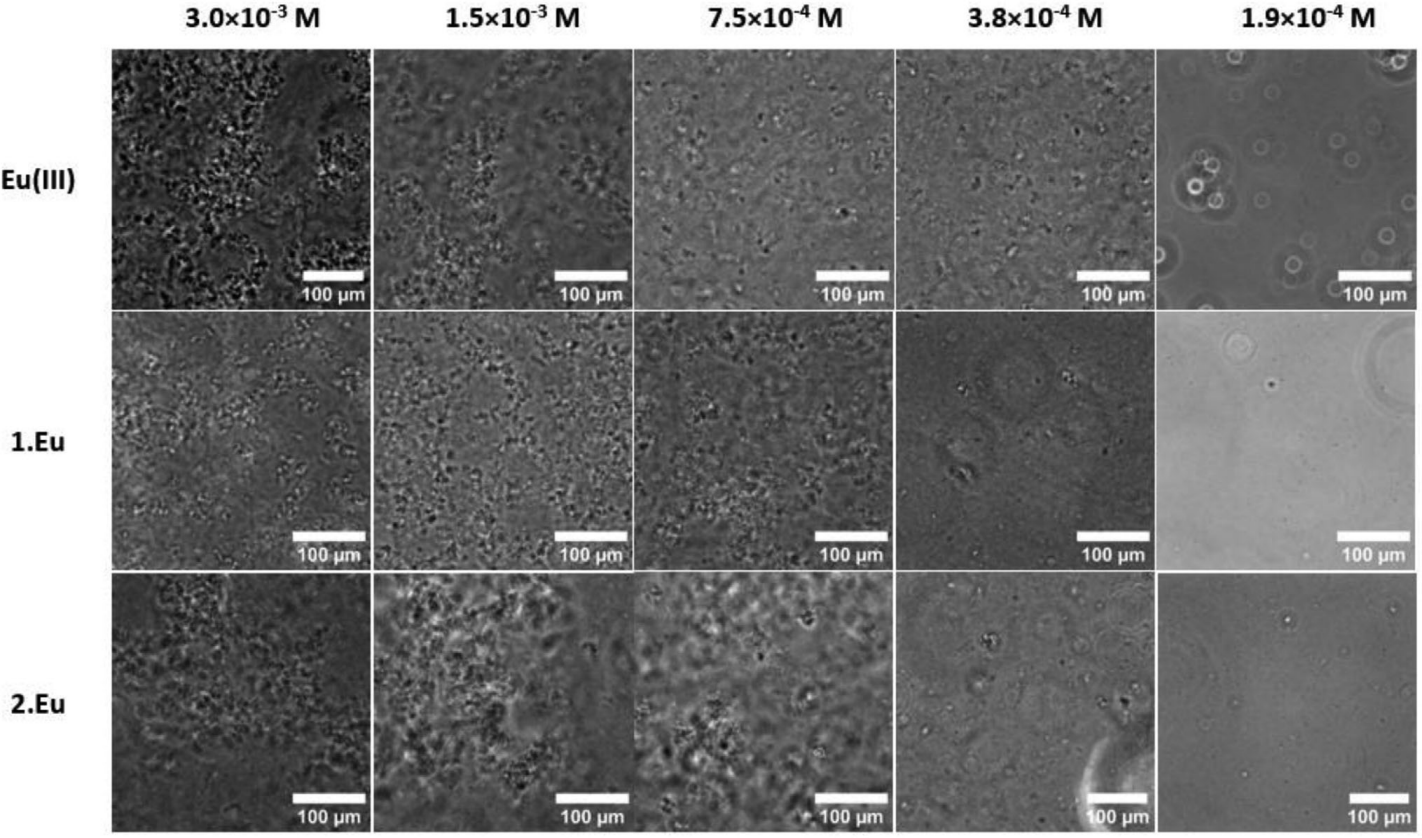
Phase‐contrast microscopy of the samples used for the microplate reader assays, where (*Top*) Eu(III), (*Middle*) **2.Eu** and (*Bottom*) **1.Eu**, were introduced, respectively, at various concentrations to RNA solutions of 1 mg/mL in aqueous HEPES (10 mM, pH 7.0)/DMSO (99:1, v/v) buffer.

### 1.7 Investigting the Binding of **1.Eu** and **2.Eu** Adenosine Monophosphate (AMP)

We observed that upon titration of RNA into the complex solutions, the absorbance spectra slowly started to present a scattering profile which, when plotted at 500 nm, roughly mimicked the shape of the emission curve at 617 nm (See Figure S14). This observation suggested that emission enhancement may have been due to changes in the “environment” of the Eu(III) complexes, and, as was mentioned above, would be felt particularly for the Δ*J* = 2 hypersensitive transition. Therefore, with a view to elucidating the reason behind the emission enhancement, titrations with small nucleotide units were undertaken to ascertain whether such emission enhancement could happen without the need for RNA‐induced aggregation.

The titrations showed that, with (mono)‐adenosine, very limited emission activation was observed, being almost none in the case of **2.Eu** and only around double for **1.Eu** (See Figure S15). In contrast, using adenosine monophosphate (AMP), used to replicate a single unit of the RNA backbone, resulted in emission enhancement, which was much greater relative to adenosine, with around a fivefold increase for both complexes at maximum (See Figure S16). This would indicate a direct binding of the Eu(III) center to the phosphate anion. Moreover, insignificant aggregation was observed in the absorption spectra when adding AMP into **2.Eu** and none was observed for **1.Eu**, which would indicate that the aggregation was not the key factor in the emission enhancement of the latter (See Figure S17), but rather the direct binding of the complexes to RNA, which gave rise to the aggregation in the RNA structure. This would suggest that the phosphate unit was binding to the metal center along with *tpy*, leading to greater emission enhancements.

## 2 Conclusion

In conclusion, we developed the Eu(III) structures **2.Eu** and **1.Eu** and shown that their Eu(III) emission was significantly enhanced in the presence of yeast RNA and **A10** (RNA), either by carrying out such investigation in solution (convectional spectroscopic titrations) or through the use of microplate reader techniques. By titrating **2.Eu** and **1.Eu** with adenosine and AMP it was concluded that binding interactions with the phosphate groups of RNA was likely involved. The mechanism of the emission enhancement was investigated, where the quantum yield of **2.Eu** was found to be higher than for **1.Eu** once bound with RNA, confirming the formation of more emissive species. The Eu(III) lifetime measurements showed the **2.Eu** was less hydrated than **1.Eu**, however upon binding with RNA both complexes gave *q* = 1, which reflected the difference in their photophysical responses upon binding to RNA. We also demonstrated that the binding to the RNA gives rise to aggregation which we observed by using both scattering and phase‐contrast and AFM imaging. These results demonstrate how (luminescent) correlation between RNA recognition and binding to responsive lanthanide complex can have major effect on the global structural outcome, that is, aggregation, of the resulting assembly, which impart, itself, can also contribute to the luminescent changes.

## Conflicts of Interest

The authors declare no conflicts of interest.

## Supporting information



The authors have cited additional references within the Supporting Information. **Supporting File 1**: chem71601‐sup‐0001‐SuppMat.pdf.

## Data Availability

The data that support the findings of this study are available from the corresponding author upon reasonable request.
