# Supplementary material for: Formation of Lanthanide Luminescent Aggregates With RNA Using Tripodal Europium [Eu(III)] BTA Self‐Assembled Complexes
Source: Chemistry. 2026 Aug 25;32(42):e71601. doi: 10.1002/chem.71601 (PMC13755171; doi:10.1002/chem.71601)
Supplement: Supplementary file 1 [file CHEM-32-e71601-s001.pdf]

Supporting Information (SI)

Formation of lanthanide luminescent aggregates with RNA using tripodal europium [Eu(III)] BTA self-assembled complexes

Tómas A. Gudmundsson, Changzhuang Bai, Grace E. Maynard, Pall Thordarson and Thorfinnur Gunnlaugsson

Materials and Methods

Synthesis

All reagents were acquired from commercial sources and used without further purification. Deuterated solvents used for NMR analysis were purchased from Apollo Ltd. HPLC grade solvents and dry dimethylformamide (DMF) were used for synthesis without further purification. Dry dichlorometane (DCM) was obtained from a PURESOLV Solvent Purification System. Water used for synthesis was deionised in a PURELAB Flex water purifier, and milliQ water was used for experiments with RNA. Silica gel 60 F254 plates were used for thin layer chromatography.

Characterisation

NMR spectra for characterisation of synthesised materials were recorded using a Bruker Spectrospin DPX-400 operating at 400 MHz for ^{1}H-NMR and at 101 MHz for ^{13}C-NMR, or an AV-600 spectrometer operating at 600 MHz for ^{1}H-NMR and at 151 MHz for ^{13}C-NMR and were carried out at 293K. Deuterated solvents were used to reference chemical shifts. ATR Infrared (IR) spectra were recorded with a PerkinElmer Spectrum One FTIR spectrometer. ESI mass spectrometry was performed using a Bruker micrOTOF-Q III interfaced to a Dionex UltiMate 3000 LC recorded over the range 100-2000 m/z. Melting points were determined using an Electrothermal IA9000 digital melting point apparatus with 1 °C/min increments. Elemental analysis (CHN) was performed using an Exeter CE440 elemental analyser with a sample size of 1.6 to 1.9 mg in a tin capsule on a Sartorius ultra microbalance. Acetanalide was used as a standard and S-benzylthiuronium chloride as a check standard.

Synthesis and Characterisation of A10

Synthesis of the homo-adenine decameric RNA **5'-AAA AAA AAA A-3'** (**A10**) was performed on a Cytiva OligoPilot^{TM} oligonucleotide synthesiser at a 100 mmol scale based on

a methodology reported in our previous work.[1] Cytiva Primer Support 5G riboA 300 resin (0.714 g, 0.214 mmol) was loaded into the synthesiser column for solid phase synthesis. Four cycles per RNA base were performed; Detritylation (dichloroacetic acid in acetonitrile (6.00 mL), Flow 300 cm/h); Coupling (RiboA (0.200 M, 2.00 equivalents), 5-(Benzylthio)-1*H*-tetrazole solution (2.00 eq), 10 min cycle time, flow rate 23.8 mL/min); Oxidation (iodine/THF/pyridine (8:1:1, v/v/v) 4.00 eq, 2 min contact time) and Capping (Cap A/Cap B_1/Cap B_2 (2:1:1, v/v/v) 3.00 mL, 1 min contact time). These cycles were completed once per base, giving a total of nine cycles.

Cleavage from the resin and deprotection of the 3'*O*-(2-cyanoethyl)-(*N*,*N*-diisopropyl) protecting group was performed in a solution of ammonia hydroxide and 40% methylamine (1:1 v/v, 10 mL). The reaction was heated at 55 °C for one hour in a sealed pressure vessel. After an hour, the reaction was cooled to room temperature and washed with water (5 mL) and ethanol (5 mL). The filtrate was collected, and ammonia hydroxide, methylamine and ethanol were removed under reduced pressure before the crude RNA was lyophilised.

Deprotection of the secondary *tert*-Butyldimethylsilyl (TBDMS) protecting group was performed by adding anhydrous DMSO (3.00 mL) and triethylamine trihydrofluoride (TEA●3HF, 3.00 mL, 18.1 mmol) and heating the reaction mixture to 65 °C for two and a half hours. *CAUTION TEA●3HF is a dangerous category 1 corrosive and toxic chemical. All work performed with this chemical was performed in accordance with the UNSW Chemistry safety guidelines on the handling of HF products.* Upon completion the reaction mixture was cooled to room temperature and the RNA was precipitated out of solution with ice cold butanol (12.0 mL, 0.131 mmol, $r = 0.81$ gcm^{-3}) and centrifuged (4000 RPM, 5 mins). This was repeated two more times to remove all impurities, leaving the crude **A-10** RNA product.

Purification of the crude **A-10** was by anion exchange chromatography on a Shimadzu LC-20AP HPLC, using a ThermoScientific DNAPac^{TM} PA-200 column. The mobile phase A was water and sodium perchlorate (10 mmol), and mobile phase B was an aqueous solution of sodium perchlorate (300 mmol). The concentration of mobile phase B was gradually increased from 1 to 55% over the course of 30 minutes, then 55% to 95% over three minutes and held at 95% for another three minutes. It was then returned to 1% for the last six minutes. The flow rate was 5 mL/min and each run was monitored via PDA detection at 220 nm and 260 nm. The crude **A-10** sample was dissolved in water (60 mL) and run at 1.5 mL injections. The fractions containing the product were collected, combined, and lyophilised. Finally, the lyophilised

product was dissolved in 30 mL of water and dialysed for 16 hours and lyophilised one last time leaving behind a white solid (264 mg, 79.6 µmol, 37%). ^{1}H-NMR (D_2O, 600 MHz) δ 8.07 – 8.01 (m, 4H), 7.99 (s, 1H), 7.93 (d, *J* = 2.0 Hz, 1H), 7.90 – 7.82 (m, 3H), 7.84 – 7.80 (m, 2H), 7.81 (s, 1H), 7.81 (s, 1H), 7.82 – 7.77 (m, 3H), 7.69 (dd, *J* = 4.9, 1.7 Hz, 2H), 7.66 – 7.62 (m, 4H), 5.81 (d, *J* = 3.8 Hz, 1H), 5.76 (d, *J* = 4.6 Hz, 1H), 5.67 (d, *J* = 4.0 Hz, 1H), 5.61 (t, *J* = 2.0 Hz, 1H), 5.58 (s, 1H), 5.59 – 5.49 (m, 5H), 5.49 (t, *J* = 2.0 Hz, 1H), 4.37 (s, 1H), 4.30 (dt, *J* = 15.0, 4.2 Hz, 2H), 4.25 (s, 2H), 4.13 – 4.02 (m, 7H), 3.80 (dd, *J* = 13.1, 2.5 Hz, 1H), 3.73 (dd, *J* = 13.0, 3.7 Hz, 1H), 3.65 (s, 5H), 2.64 (s, 1H). ^{31}P-NMR (D_2O, 600 MHz) δ -0.80, -1.07. HRMS $(ESI)^+$ MALDI-MS m/z: $[M + 5H]^+$ calculated for $C_{100}H_{127}N_{50}O_{58}P9$: 3234.62, found: 3234.42.

Absorbance and Luminescence Assay

All samples including blanks were prepared in triplicate in a 384 well plate, 20 minutes before starting the measurement. A 25 µL solution of RNA was pipetted into the relevant well followed by 25 µL of complex solution in an aqueous HEPES (10 mM, pH 7.0)/DMSO (99:1, v/v) buffer. Blanks solutions were prepared in triplicate as well and subtracted from the relevant spectra for analysis. Absorbance was recorded from 300 to 700 nm. Concentrations were chosen in order to avoid signal saturation while maintaining a high concentration for aggregation. Luminescence was recorded from 570 to 720 nm to monitor the characteristic phosphorescence emission from Eu(III). Measurements with a 1:1 complex:RNA mass ratio only taken at a single wavelength: 700 nm for absorbance to monitor scattering, and 617 nm for luminescence to monitor the λ_{max} of the Eu(III)-centred emission.

Microscopy

Phase-Contrast Microscopy images were acquired on a Nikon ECLIPSE TE2000-U Microscope with a CoolLED *p*T-100 white light source with exposure set to 5 ms, binning to 2 and shutter set to pE300 on 40x magnification. Samples were prepared by mixing 5 µL of RNA solution with 5 µL complex or other relevant solution directly on a glass plate. All solutions were in an aqueous HEPES (10 mM, pH 7.0)/DMSO (99:1, v/v) buffer. Image processing was performed with ImageJ. Atomic Force Microscopy (AFM) imaging was carried out on a Bruker Dimension ICON SPM using PeakForce tapping mode in air. The silicon tip on a nitride lever Bruker SCANASYST-AIR model was used. The tip had a length of 115 µm, a width of 25 µm, and the spring constant 0.4 N/m. Freshly prepared samples (20 µL) were deposited onto a freshly cleaved mica surface for 5 min, followed by wicking off most of the

liquid from the mica surface using filter paper. After excess sample was wicked off, Milli-Q water (50 μL) was deposited on the mica for 30 s to absorb any remaining HEPES buffer. The washing process was repeated three times, the samples then were allowed to dry at room temperature for overnight before imaging.

Photophysical Measurements

All photophysical measurements involving titrations with RNA, adenosine or AMP were performed on an Edinburgh Instruments FS5 Spectrofluorometer. Samples in an aqueous HEPES (10 mM, pH 7.0)/DMSO (99:1, v/v) buffer with a volume of 2000 μL were prepared in quartz cuvettes, and dilution upon titration never exceeded 1% of the initial volume. Dilution corrections were applied during data processing. Transmission scans were converted to absorbance after measurements. All emission scans were repeated in triplicate, and transmission and excitation scans in duplicate. All measurements were performed with a standard SC-05 single cuvette sample holder module, except quantum yield measurement which were performed with the SC-30 Integrating Sphere module. Fitting Hill curves was achieved using a custom R program.

The compositional analysis of the complex solutions was performed using a Cary 60 UV-Vis spectrophotometer for absorbance measurements, and a Gary Eclipse Fluorescence Spectrophotometer was used for luminescence spectra. Stock solution of the ligands were prepared at 3.0×10^{-5} M for **1** and 1.0×10^{-5} M for **2** by dissolving the ligands in DMSO and diluting in HEPES (10 mM, pH 7.0) buffer solution, creating an aqueous HEPES (10 mM, pH 7.0)/DMSO (99:1, v/v) buffer. The changes in the absorbance spectra were fit to ML species by non-linear regression analysis with the aid of the ReactLab analysis software. The absorbance measurements returned good fits for both **1** and **2**, and therefore were repeated in triplicate to provide the reported stability constants.

Synthesis and Characterisation of Ligand 1

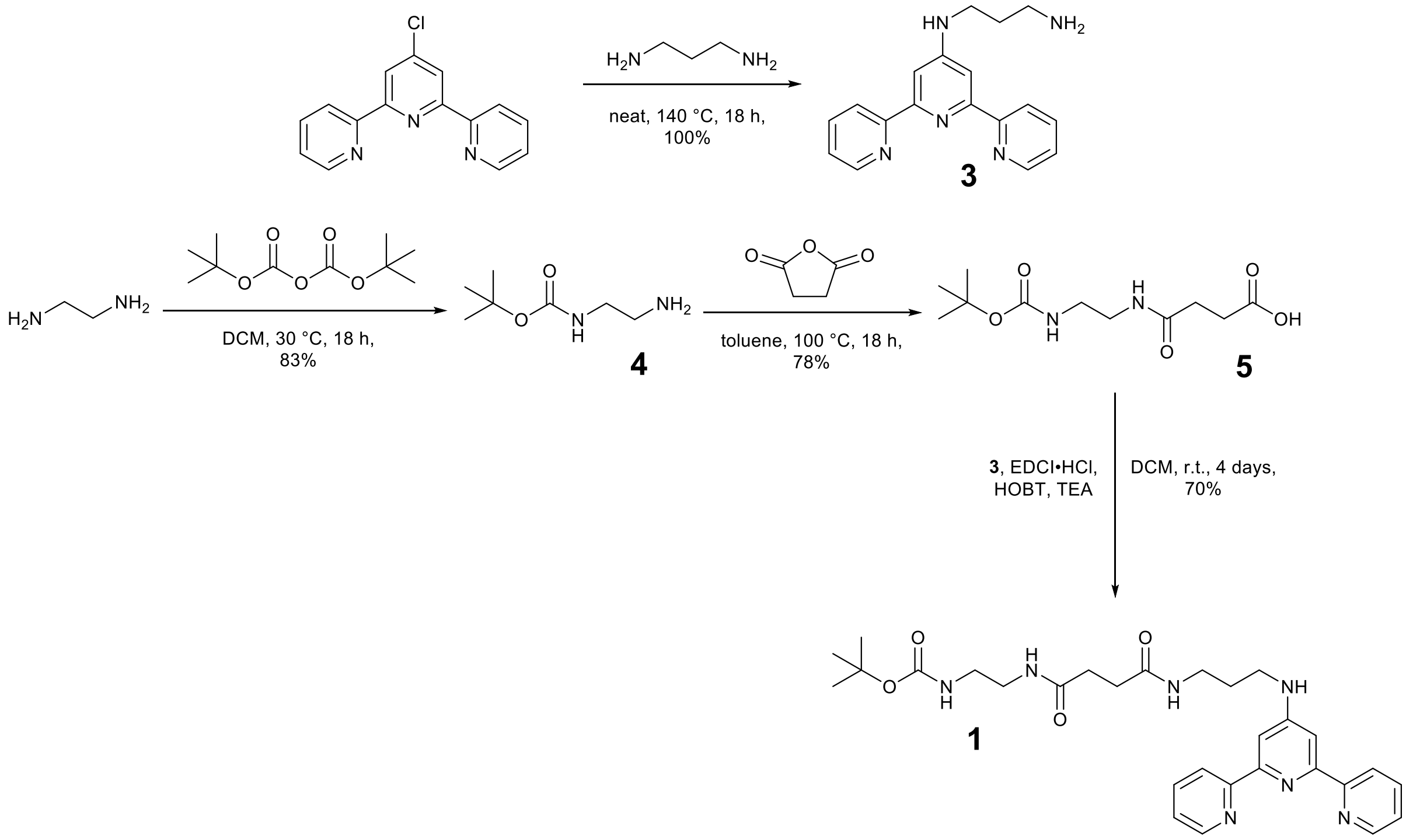

Scheme S1: Synthesis of ligand **1**.

***N^1^*-([2,2':6',2''-Terpyridin]-4'-yl)propane-1,3-diamine** (**3**) **3** was prepared according to literature procedure.[2] 4′-Chloro-2,2′:6′,2″-terpyridine (681 mg, 2.54 mmol) was suspended in 1,3-diaminopropane (2.5 mL, 30 mmol, 12 eq.), and left to reflux at 140 °C overnight. Subsequently, the solution was precipitated out in ice-cold H_2O (200 mL). The precipitate was filtered and washed with H_2O and left to dry overnight with P_2O_5 in a dessicator. The product was isolated as a pale-yellow powder **3** (774 mg, 100%). ^{1}H-NMR (600 MHz, DMSO) δ 8.66 (ddd, *J* = 4.7, 1.4, 0.9 Hz, 2H, CH_{tpy}), 8.55 (m, 2H, CH_{tpy}), 7.95 (m, 2H, CH_{tpy}), 7.67 (s, 2H, CH_{tpy}), 7.43 (ddd, *J* = 7.5, 4.8, 1.4 Hz, 2H, CH_{tpy}), 6.89 (t, *J* = 5.2 Hz, 1H, NH), 3.26 (m, 2H, CH_2), 2.68 (t, *J* = 6.7 Hz, 2H, CH_2), 1.67 (m, 2H, CH_2), 1.52 (s, 2H, NH_2). ^{13}C NMR (151 MHz, DMSO) δ 156.01 (q), 155.62 (q), 154.84, 148.90, 136.98, 123.79, 120.56, 103.60 (q), 40.03, 39.97, 32.33. m.p. 145.4 – 147.2 °C (lit. 148 °C).[3] ESI-HRMS *(m/z)* calcd. for $C_{18}H_{19}N_5$ 305.1640 found $[M+H]^+$ 306.1713.

***tert*-Butyl (2-aminoethyl)carbamate** (**4**) The synthesis of **4** was adapted from literature.[4] Di-*tert*-butyl dicarbonate (2381 mg, 10.9 mmol) was added to a dropping funnel with DCM (50 mL) and dropped into a solution of ethylenediamine (7.3 mL, 110 mmol, 10 eq.) in DCM (50

mL) at 0 °C for 2 hours. The solution was left to stir at 30 °C overnight. The reaction mixture was extracted out of sat. $NaHCO_3$ three times with DCM, dried with $MgSO_4$ and the solvent evaporated under reduced pressure. The remaining oil was dried further under high vacuum. The product was isolated as a clear yellow oil **4** (1446 mg, 83%). ^{1}H NMR (600 MHz, DMSO) δ 6.74 (s, 1H, CONH), 2.90 (app. q, 2H, CH_2), 2.52 (t, J = 6.3 Hz, 2H, CH_2), 1.37 (s, 9H, $C(CH_3)_3$). ^{13}C NMR (151 MHz, DMSO) δ 156.15 (q), 77.86 (q), 44.06, 42.03, 28.71. ESI-HRMS *(m/z)* calcd. for $C_7H_{16}N_2O_2$ 160.1212 found $[M+H]^+$ 161.1285. ν_{max} (ATR) cm^{-1}: 3377m (N-H), 2991w, 2970w, 2929w, 1680s (C=O), 1519s, 1461w, 1449m, 1385w, 1375m, 1321w, 1280s, 1259m, 1233s, 1145s, 972s, 866m, 798w, 776m, 759w, 701w, 585m.

4-((2-((*tert*-Butoxycarbonyl)amino)ethyl)amino)-4-oxobutanoic acid (**5**) The synthesis of **5** was adapted from literature.[5] Succinic anhydride (757 mg, 7.56 mmol, 1.1 eq.) and **4** (1075 mg, 6.71 mmol) were suspended in toluene (120 mL) and left to reflux for 4 hours. The suspension was then left to cool to room temperature, vacuum filtered and collected as a white powder **5** (1361 mg, 78%).[6] ^{1}H NMR (400 MHz, DMSO) δ 12.06 (s, 1H, COOH), 7.85 (t, J = 4.9 Hz, 1H, CONH), 6.76 (t, J = 5.3 Hz, 1H, CONH), 3.04 (dq, J = 6.1 Hz, 4.9 Hz, 3H, CH^2), 2.95 (dq, J = 6.1 Hz, 5.3 Hz, 2H, CH_2), 2.41 (t, J = 7.0 Hz, 3H, CH_2), 2.29 (t, J = 7.0 Hz, 2H, CH_2), 1.37 (s, 9H, $C(CH_3)_3$). ^{13}C NMR (101 MHz, DMSO) δ 173.89 (q), 171.15 (q), 155.65 (q), 77.70 (q), 39.63, 38.78, 30.06, 29.17, 28.25. m.p. 132.2 – 133.6 °C .ESI-HRMS *(m/z)* calcd. for $C_{11}H_{20}N_2O_5$ 260.1372 found $[M+Na]^+$ 283.1264. ν_{max} (ATR) cm^{-1}: 3352m (N-H), 3321m (N-H), 2984w, 2962w, 2923w, 2891b (O-H), 1700m (C=O), 1667s (C=O), 1640s (C=O), 1552s, 1514s, 1444w, 1412m, 1383m, 1369m, 1342w, 1313w, 1272s, 1275s, 1220s, 1139s, 1032w, 969m, 904m, 850m, 771w, 684m, 627m, 556m.

***tert*-Butyl (2-(4-((3-([2,2':6',2''-terpyridin]-4'-ylamino)propyl)amino)-4-oxobutanamido)ethyl)carbamate** (**1**) **5** (1361 mg, 5.23 mmol) and EDCI·HCl (1502 mg, 7.84 mmol, 1.5 eq.) were dissolved in dry DCM (120 mL). The solution was cooled to 0 °C and triethylamine (1.10 mL, 7.91 mmol, 1.5 eq) was added and left to stir for 10 minutes. HOBt (921 mg, 6.82 mmol, 1.3 eq.) and **3** (1672 mg, 5.47 mmol, 1.1 eq.) were added, and the solution was left to stir at room temperature for 4 days with a $CaCl_2$ drying tube. The solution was washed twice with sat. $NaHCO_3$ solution, four times with 10% K_2CO_3, dried over $MgSO_4$ and the solvent evaporated under reduced pressure. The resulting solid was dissolved in DCM, precipitated in diethyl ether and filtered to yield a pale-yellow solid **1** (2005 mg, 70%). ^{1}H

NMR (600 MHz, DMSO) δ 8.80 (d, *J* = 4.7 Hz, 2H, CH_{tpy}), 8.69 (d, *J* = 7.9 Hz, 2H, CH_{tpy}), 8.08 (m, 2H, CH_{tpy}), 8.07 (t, *J* = 6.5 Hz , 1H, CONH), 7.96 (t, *J* = 5.2 Hz, 1H, CONH), 7.80 (s, 2H, CH_{tpy}), 7.57 (m, 2H, CH_{tpy}), 6.98 (t, *J* = 5.2 Hz, 1H, CONH), 6.88 (t, *J* = 5.3 Hz, 1H, NH), 3.36 (app. q, 2H, CH_2), 3.31 (app. q, 2H, CH_2), 3.17 (app. q, 2H, CH_2), 3.08 (app. q, 2H, CH_2), 2.45 (m, 4H, CH_2), 1.87 (p, *J* = 6.8 Hz, 2H, CH_2), 1.49 (s, 9H, $C(CH_3)_3$). ^{13}C NMR (151 MHz, DMSO) δ 171.60 (q), 171.48 (q), 156.01 (q), 155.65, 155.58 (q), 154.95, 148.97, 137.08, 123.89, 120.66, 103.75 (q), 77.69 (q), 40.05, 39.25, 38.76, 36.42, 30.90, 30.86, 28.57, 28.25. m.p. 126.7 – 130.4 °C. Anal. Calcd. for $C_{29}H_{37}N_7O_4{\cdot}0.8H_2O$: C, 61.97; H, 6.92; N, 17.44. Found: C, 62.08; H, 6.76; N, 17.36 ESI-HRMS *(m/z)* calcd. for $C_{29}H_{37}N_7O_4$ 547.2907 found $[M+H]^+$ 548.2980. ν_{max} (ATR) cm^{-1}: 3337m (N-H), 3306m (N-H), 3060w, 2977w, 2933w, 2868w, 2160w, 2015w, 1681s (C=O), 1630s (C=O), 1610m, 1584s, 1524s, 1440m, 1403m, 1367m, 1333w, 1274m, 1244m, 1163s, 1090w, 1041w, 977m, 851w, 788s, 726m, 655m, 618m, 566m.

Synthesis and Characterisation of Ligand 2 (This has previously been communicated and is only included here for the reader’s convivence)

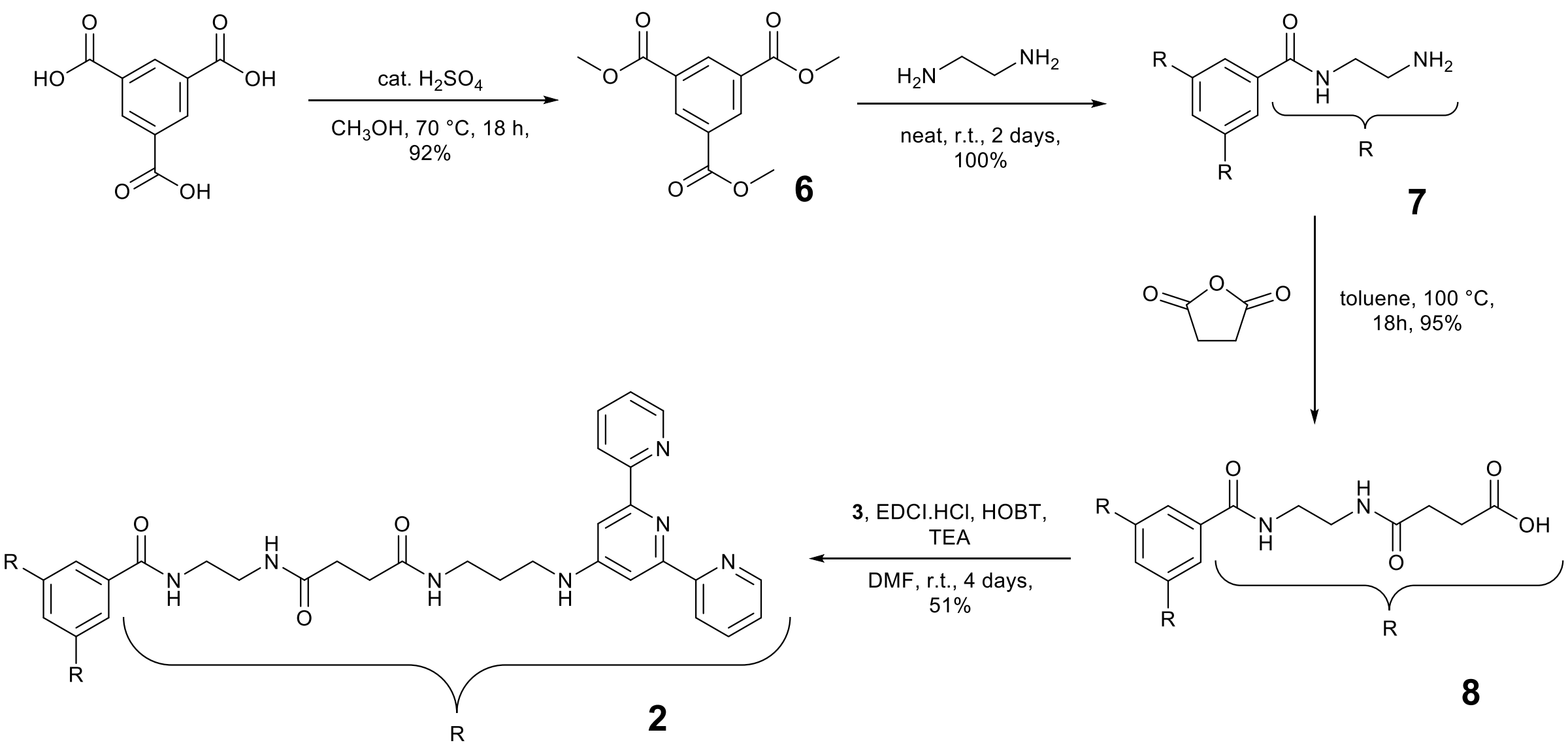

Scheme 2: Synthesis of ligand **2**.

Trimethyl benzene-1,3,5-tricarboxylate (6) The synthesis of **6** was adapted from literature.[7] Trimesic acid (1980 mg, 9.424 mmol) was dissolved in methanol (30 mL) before adding conc. H_2SO_4 (1.5 mL) and refluxing overnight. The reaction mixture was cooled on ice and filtered to yield a crystalline white powder (3011 mg, 95%). ^{1}H-NMR (400 MHz, $CDCl_3$) δ 8.86 (s, 3H, CH), 3.98 (s, 9H, CH_3). m.p. 146 – 149 °C. ESI-HRMS *(m/z)* calcd. for $C_{12}H_{12}O_6$ 252.0634 found $[M+H]^+$ 253.0710. ν_{max} (ATR) cm^{-1}: 3100w, 2958w, 1725s (C=O), 1613w, 1447w, 1432m, 1341w, 1238s, 1140m, 1101m, 996s, 928m, 883w, 873w, 802w, 734s, 721s.

N^1^ —

N^1,N^3,N^5-tris(2-Aminoethyl)benzene-1,3,5-tricarboxamide (7) **6** (384 mg, 1.52 mmol) was dissolved in neat ethane-1,2-diamine (5 mL) and stirred for 2 days at ambient temperature. The mixture was then concentrated under reduced pressure, before evaporating azeotropically using H_2O followed by toluene, and dried under high vacuum. The product was obtained as a yellow oil (512 mg, 100%). ^{1}H-NMR (400 MHz, DMSO) δ 8.62 (t, *J* = 5.3 Hz, 3H, CONH), 8.39 (s, 3H, CH_{Ar}), 3.29 (app q., 6H), 2.70 (t, *J* = 6.5 Hz, 6H). $C_{15}H_{24}N_6O_3$ 336.1910 found $[M+H]^+$ 337.1990. ν_{max} (ATR) cm^{-1}: 3284m (broad) (N-H), 3071m, 2935m, 2873m, 1641s (C=O), 1591m, 1537s (C=O), 1432m, 1288s, 1186m, 1109w, 913w, 820w, 703m.

4,4',4''-((((Benzene-1,3,5-tricarbonyl)tris(azanediyl))tris(ethane-2,1-diyl))tris(azanediyl)) tris(4-oxobutanoic acid) (8) Toluene (50 mL) was added to **7** (723 mg,

1.97 mmol) and succinic acid anhydride (621 mg, 6.21 mmol, 3.2 eq.), and the mixture was refluxed overnight. The suspension was filtered while hot and the remaining precipitate was washed with warm toluene before leaving to dry under vacuum. The product was collected as a white precipitate (1191 mg, 95%). ^{1}H-NMR (400 MHz, DMSO) δ 12.06 (s, 3H, COOH), 8.71 (t, J = 5.5 Hz, 3H, CONH), 8.42 (s, 1H, CH_{Ar}), 8.00 (t, J = 5.6 Hz, CONH), 3.33 (app. q, 6H, CH_2), 3.24 (app. q, 6H, CH_2), 2.44 (t, J = 7.1 Hz, 6H, CH_2), 2.33 (t, J = 7.1 Hz, 6H, CH_2). m.p. 179 – 187 °C. $C_{27}H_{36}N_6O_{12}$ 636.2391 found $[M]^-$ 635.2316. ν_{max} (ATR) cm^{-1}: 3291m (N-H), 3076m, 2938m, 2624w (broad) (O-H), 1716m (C=O), 1634s (C=O), 1532s, 1430m, 1236s, 1171m, 1111m, 915w, 833w, 682m.

***N^1,N^3,N^5*-tris(2-(4-((3-([2,2':6',2''-Terpyridin]-4'-ylamino)propyl)amino)-4-oxobutanamido)ethyl)benzene-1,3,5-tricarboxamide (2)** The synthesis was performed similarly to ligand **1**. **8** (227 mg, 0.357 mmol) and EDCI·HCl (1371 mg, 7.152 mmol, 20 eq.) were dissolved in dry DMF (25 mL). The solution was cooled to 0 °C and triethylamine (1.0 mL, 7.2 mmol, 20 eq.) was added and left to stir for 10 minutes. HOBt (197 mg, 1.46 mmol, 4.1 eq.) and **3** (349 mg, 1.14 mmol, 3.2 eq.) were added, and the solution was left to stir at room temperature for 4 days with a $CaCl_2$ drying tube. The solution was washed twice with sat. $NaHCO_3$ solution, four times with 10% K_2CO_3, dried over $MgSO_4$ and the solvent evaporated under reduced pressure. The resulting solid was dissolved in DCM/MeOH, precipitated in diethyl ether and filtered to yield a pale-yellow solid (273 mg, 51%). ^{1}H-NMR (600 MHz, DMSO) δ 9.44 (s, 3H, CH_{Ar}), 8.66 (d, J = 4.3 Hz, 6H, CH_{tpy}), 8.56 (d, J = 7.9 Hz, 6H, CH_{tpy}), 8.08 – 7.98 (m, 6H, CONH), 7.94 (td, J = 7.8, 1.8 Hz, 6H, CH_{tpy}), 7.67 (s, 6H, CH_{tpy}), 7.46 – 7.39 (m, 3H, CH_{tpy}), 6.91 (t, J = 5.2 Hz, 6H, NH), 3.26 – 3.14 (m, 24H, CH_2), 2.39 – 2.28 (m, 12H, CH_2), 1.75 (p, J = 6.8 Hz, 2H, CH_2). ^{13}C-NMR (151 MHz, DMSO) δ 171.88, 171.64, 171.49, 171.41, 156.00, 155.56, 154.92, 148.95, 137.05, 123.86, 120.63, 104.10, 64.93, 40.06, 37.42, 36.40, 30.92, 30.86, 30.76, 28.55. m.p. 234 – 238 °C. Anal. Calcd. for $C_{81}H_{87}N_{21}O_9 \cdot 8H_2O$: C, 59.22; H, 6.32; N, 17.90. Found: C, 59.00; H, 5.60; N, 17.50. HRMS *(m/z)* calcd. for $C_{81}H_{87}N_{21}O_9$ 1497.6996 found $[M+2H]^{2+}$ 749.8571. ν_{max} (ATR) cm^{-1}: 3288m (N-H), 3072w, 2937w, 1643s (C=O), 1606s, 1583s, 1564m, 1538s, 1464m, 1441m, 1406m, 1360m, 1261m, 1227m, 1096w, 1043w, 985m, 857w, 792s, 735w, 693w, 659w.

Figures

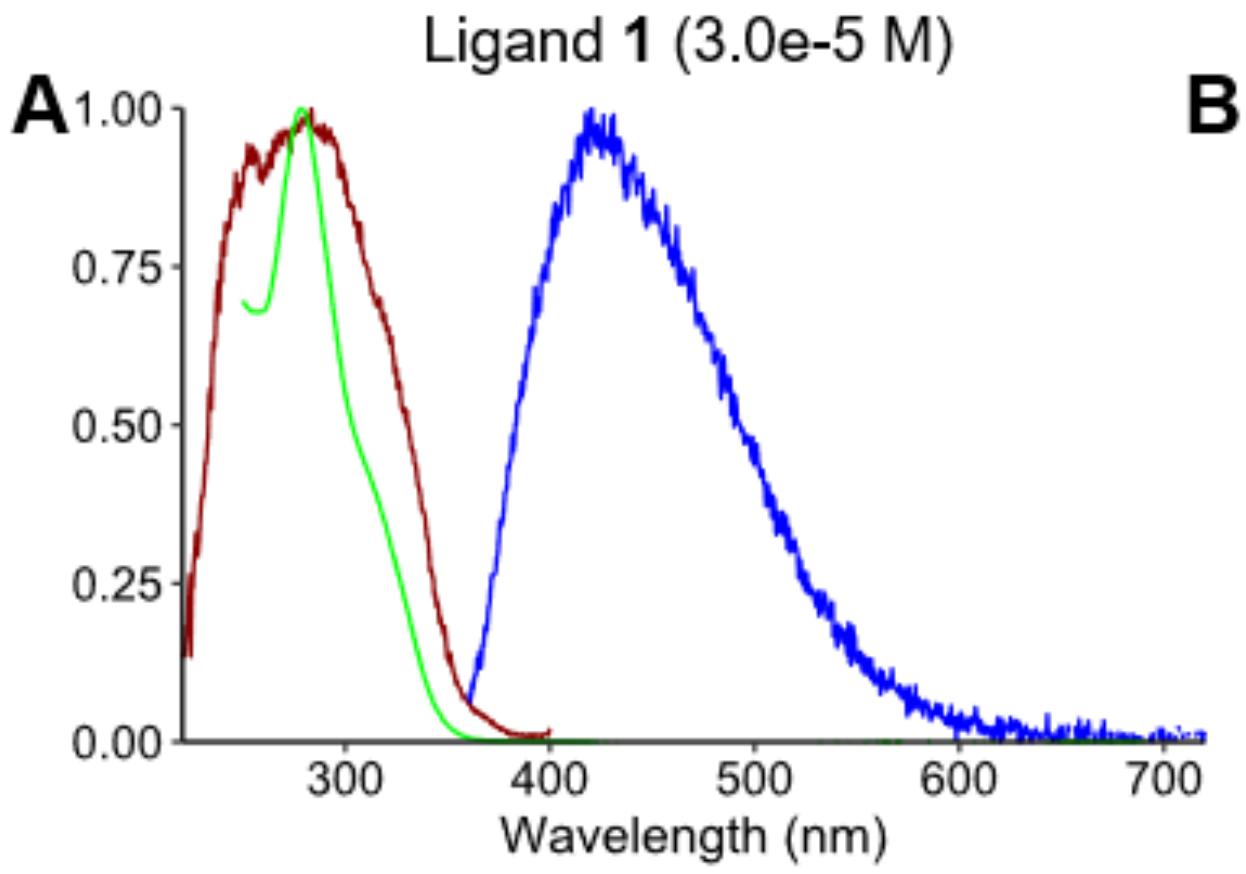

Figure S1: Normalised absorbance, emission, and excitation spectra of the ligands in in aqueous HEPES (10 mM, pH 7.0)/DMSO (99:1, v/v) buffer. A) Ligand **1** (3.0×10^{-5} M). A) Ligand **2** (1.0×10^{-5} M).

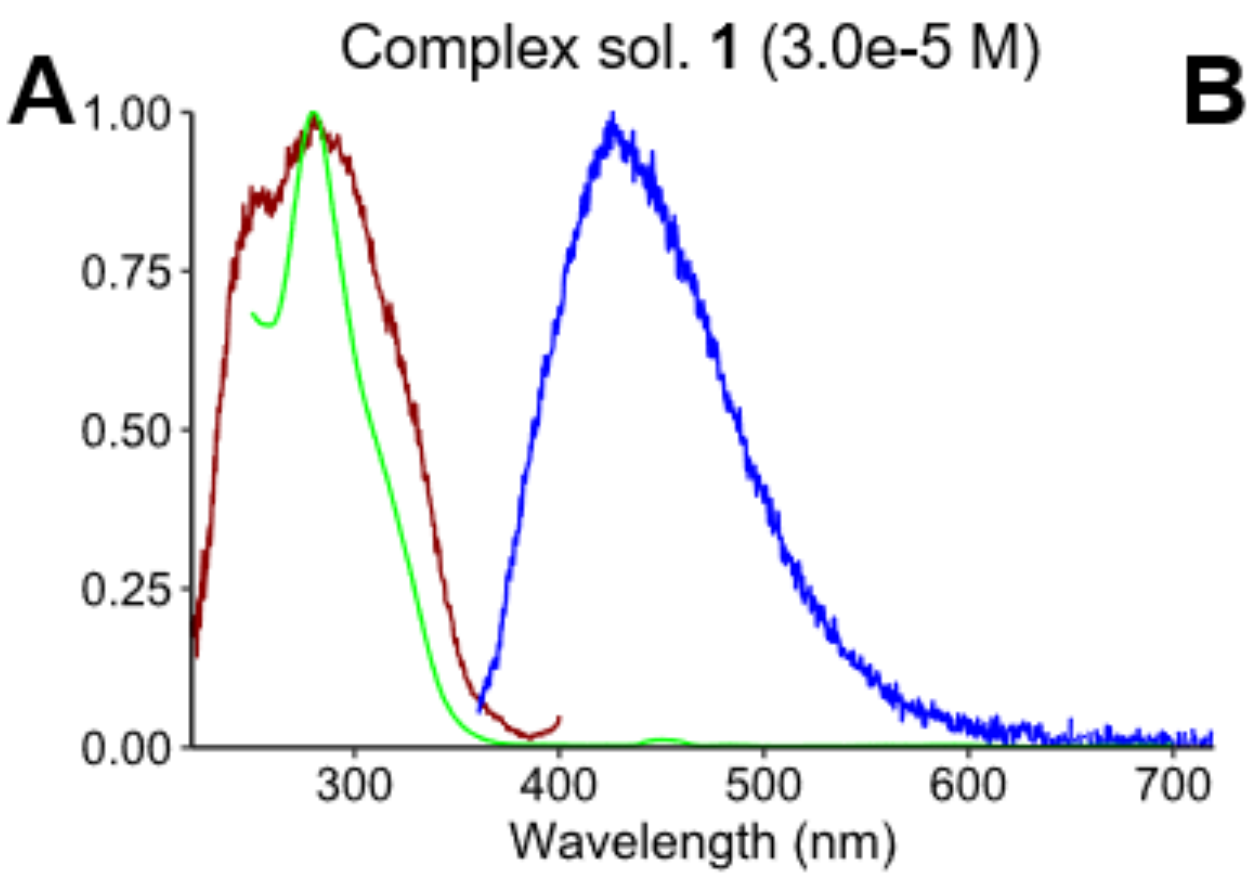

Figure S2: Normalised absorbance, emission, and excitation spectra of the complex solutions in in aqueous HEPES (10 mM, pH 7.0)/DMSO (99:1, v/v) buffer. A) Solution **1.Eu** (3.0×10^{-5} M). A) Solution **2.Eu** (1.0×10^{-5} M).

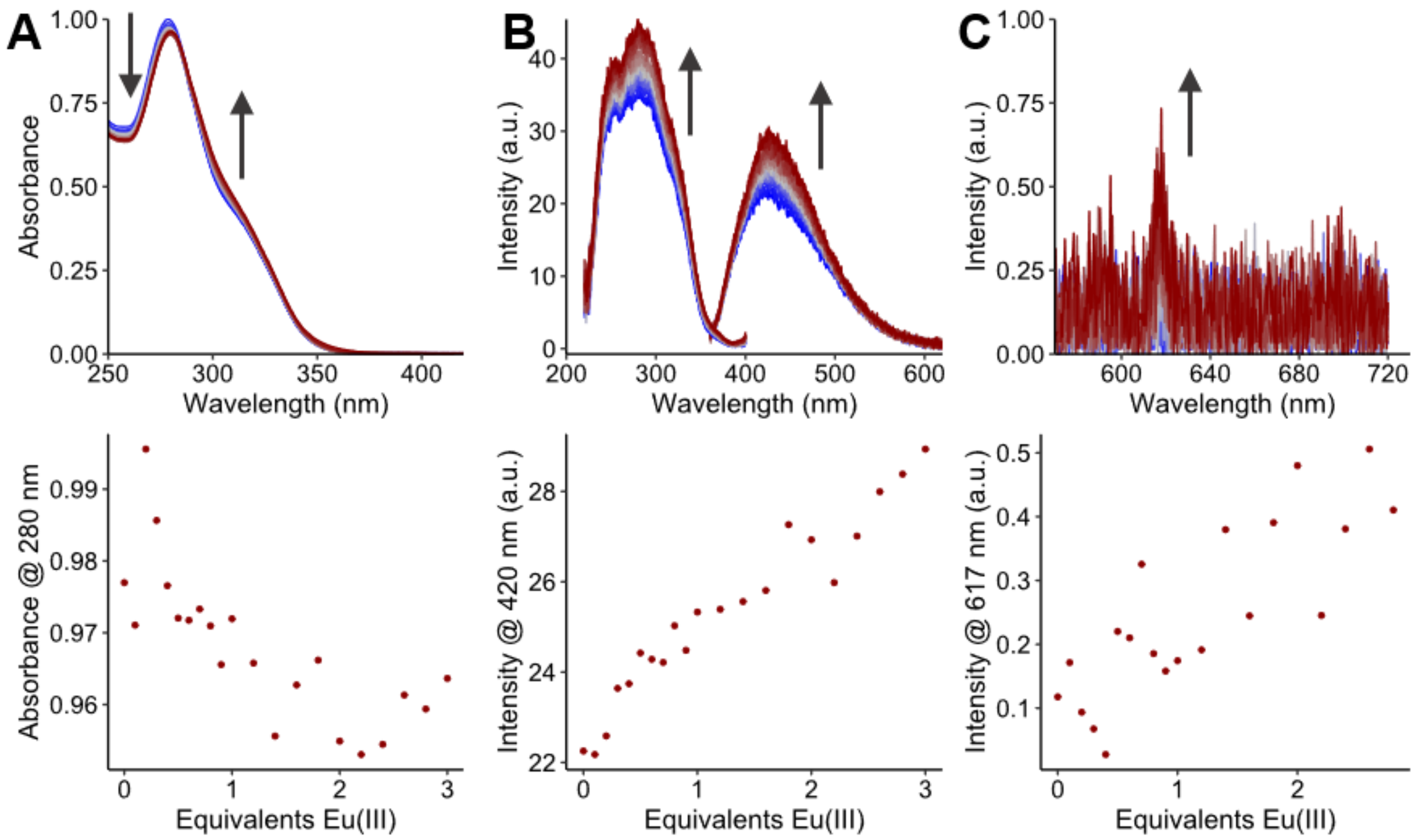

Figure S3: Titration of **1** (C = 1.0×10^{-5} M) with 0 → 3.0 equivalents $EuCl_3$ in in aqueous HEPES (10 mM, pH 7.0)/DMSO (99:1, v/v) buffer where the full spectra and binding isotherms are presented for: A) Absorbance, B) Fluorescence emission (λem = 320 nm) and excitation (λem = 420 nm), and C) Phosphorescence emission (λex = 320 nm).

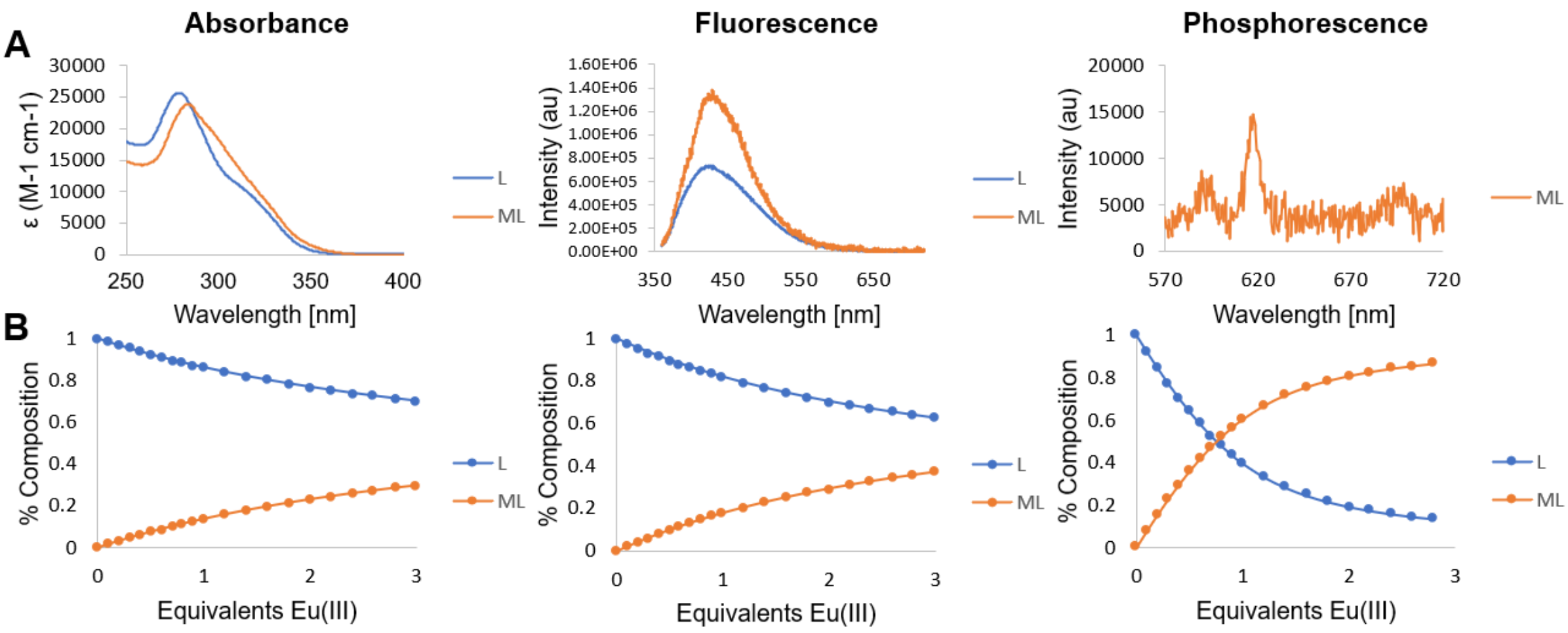

Figure S4: Non-linear regression analysis of the absorbance, fluorescence, and phosphorescence titration results with **1** (3.0×10^{-5} M) and Eu(III) from **Figure S8** acquired from ReactLab. A) Re-calculated spectra of ML species. B) Speciation diagrams for the titration points across experiments.

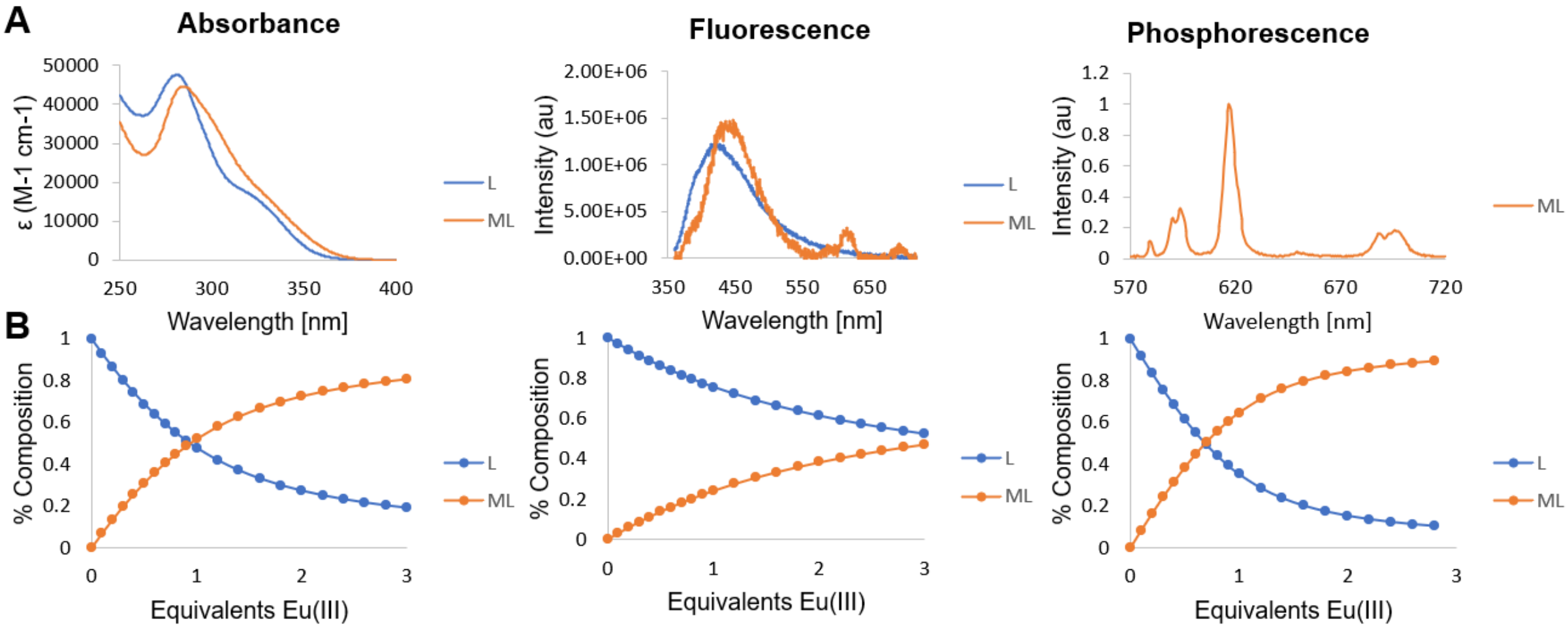

Figure S5: Non-linear regression analysis of the absorbance, fluorescence, and phosphorescence titration results with **2** (1.0×10^{-5} M) and Eu(III) from **Figure S9** acquired from ReactLab. A) Re-calculated spectra of ML species. B) Speciation diagrams for the titration points across experiments.

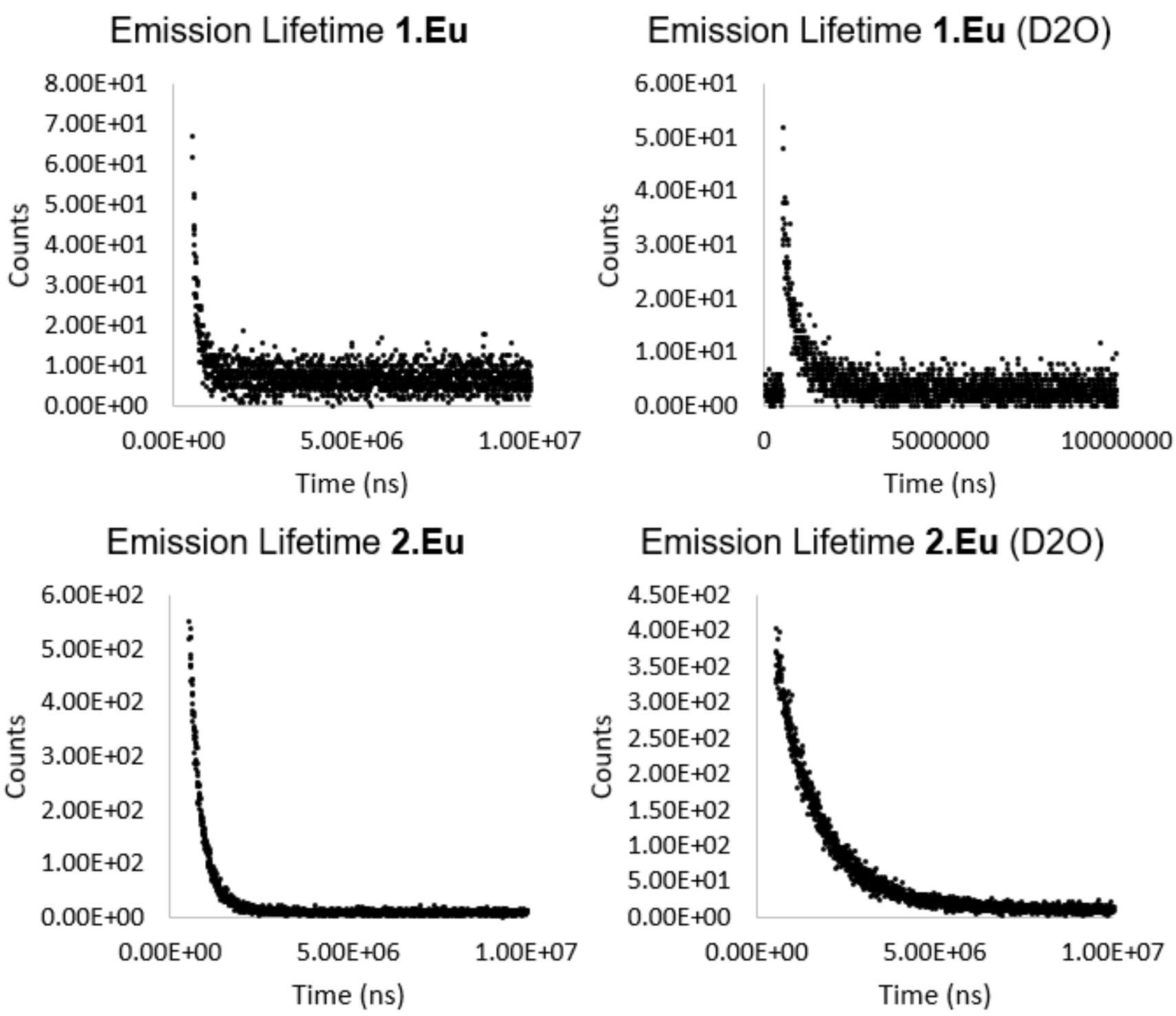

Figure S6: Phosphorescence mission lifetime measurements at 617 nm (λ_{ex} = 320 nm) with **1.Eu** (C = 3.0×10^{-5} M) and **2.Eu** (C = 1.0×10^{-5} M) in H_2O and D_2O based aqueous HEPES (10 mM, pH 7.0)/DMSO (99:1, v/v) buffers.

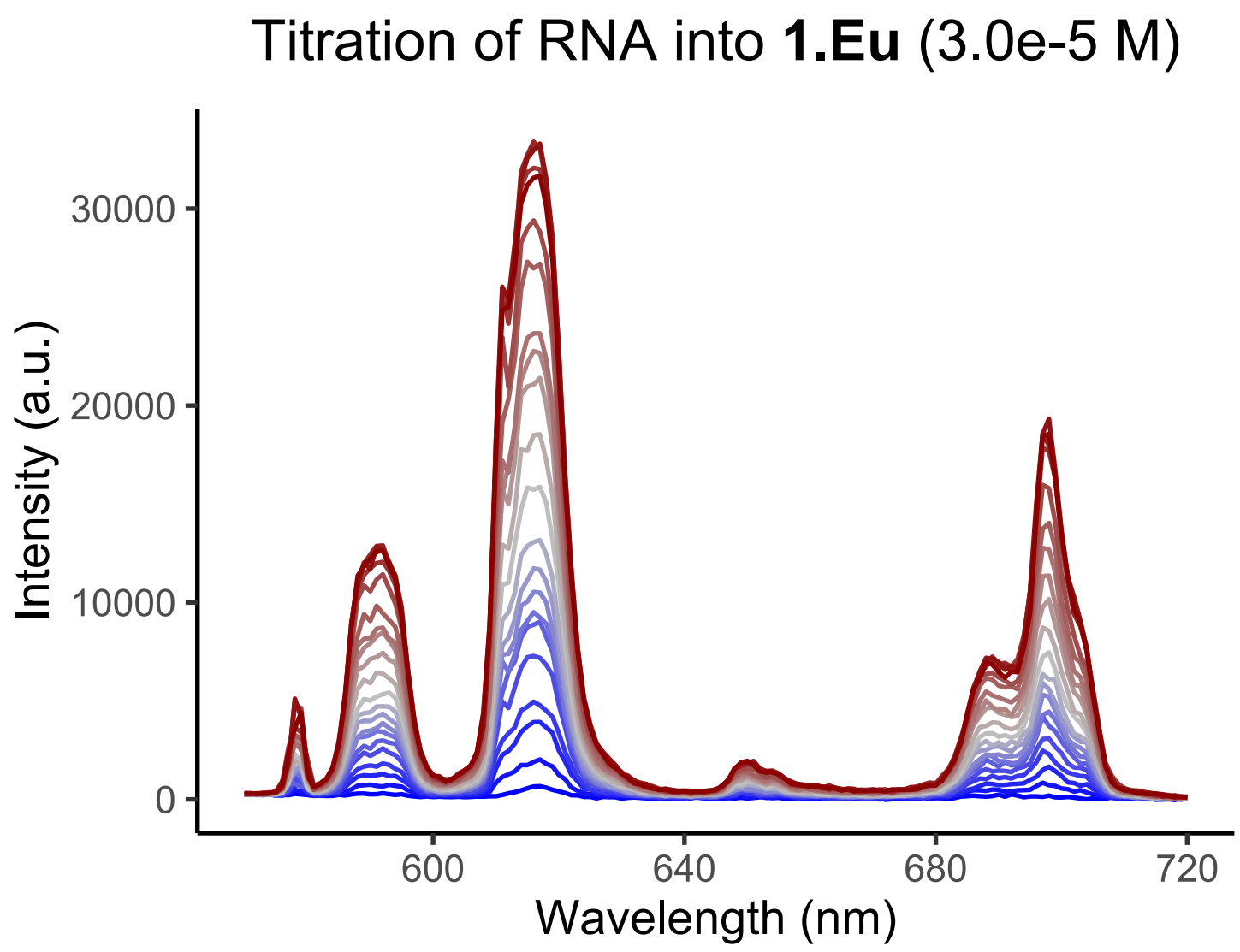

Figure S7: Full phosphorescence emission data from the addition of RNA to **1.Eu** in aqueous HEPES (10 mM, pH 7.0)/DMSO (99:1, v/v) buffer.

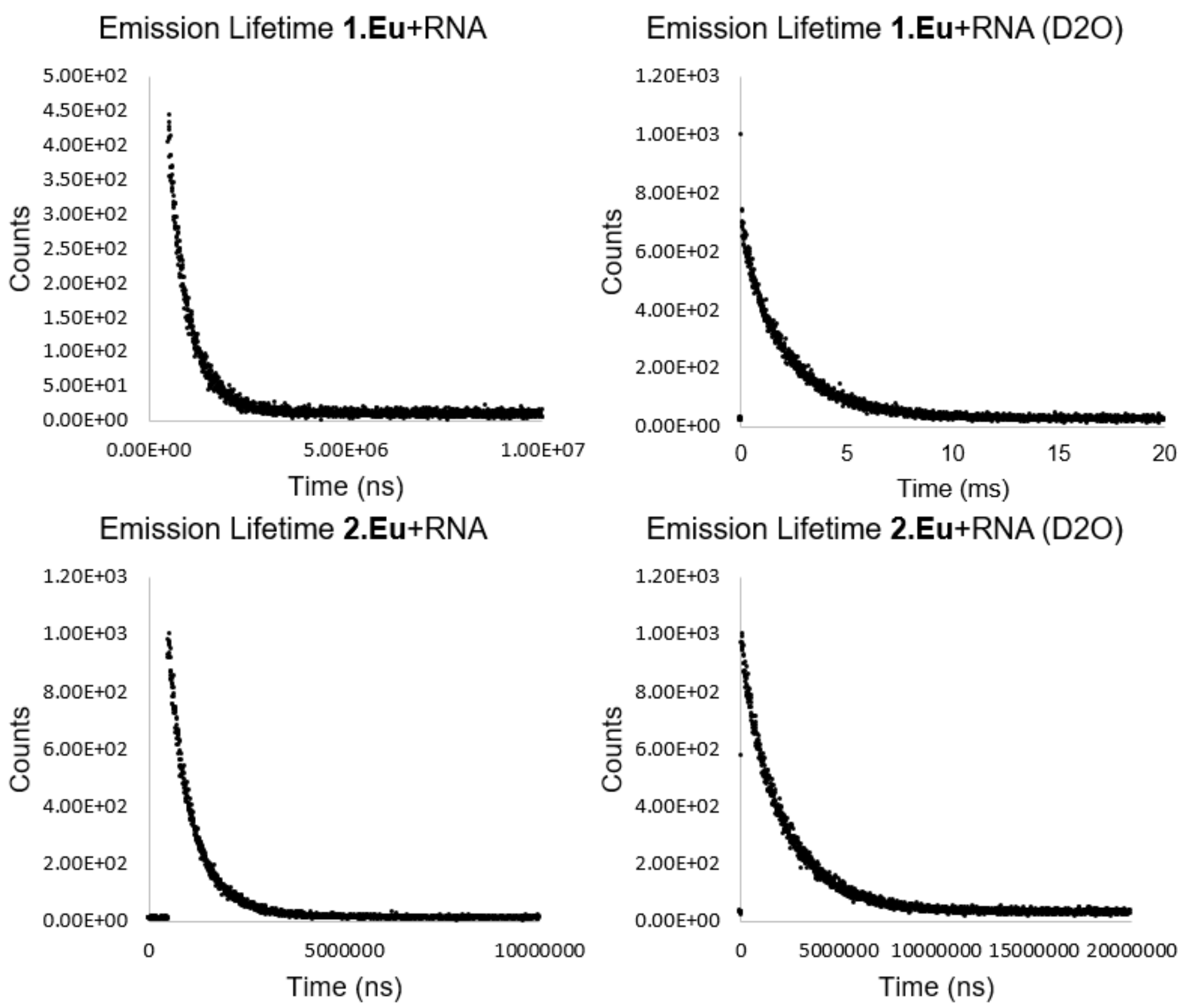

Figure S8: Phosphorescence mission lifetime measurements at 617 nm (λ_{ex} = 320 nm) with **1.Eu** (C = 3.0×10^{-5} M) and **2.Eu** (C = 1.0×10^{-5} M) saturated with RNA in H_2O and D_2O aqueous HEPES (10 mM, pH 7.0)/DMSO (99:1, v/v) buffer

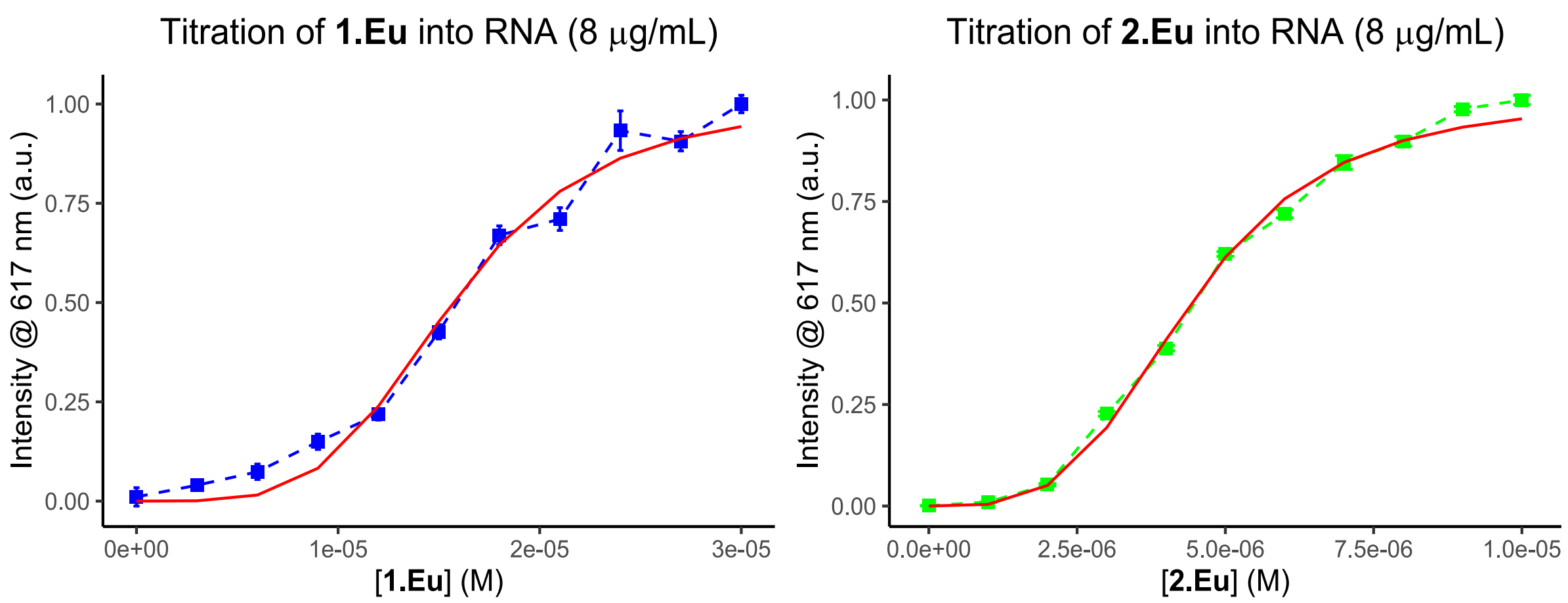

Figure S9: Titration of fixed amounts of 8 μg/mL RNA into solutions of **1.Eu** (left) and **2.Eu** (right) at various concentrations in aqueous HEPES (10 mM, pH 7.0)/DMSO (99:1, v/v) buffer. The red line represents the curve fit to the Hill equation (**Equation S2**).

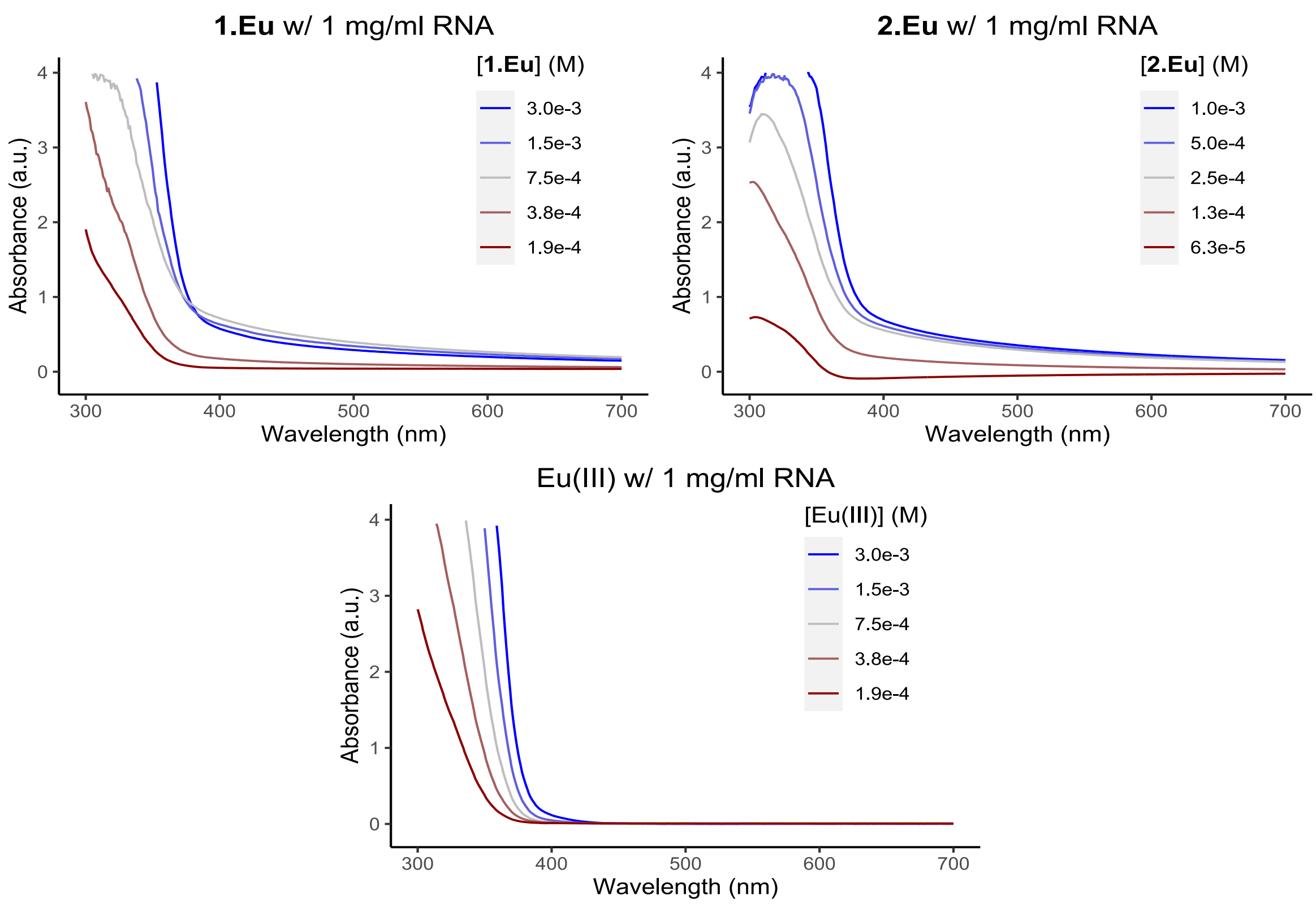

Figure S10: Absorbance plate reader assay for various concentrations of **1.Eu**, **2.Eu** and Eu(III) mixed with RNA in aqueous HEPES (10 mM, pH 7.0)/DMSO (99:1, v/v) buffer.

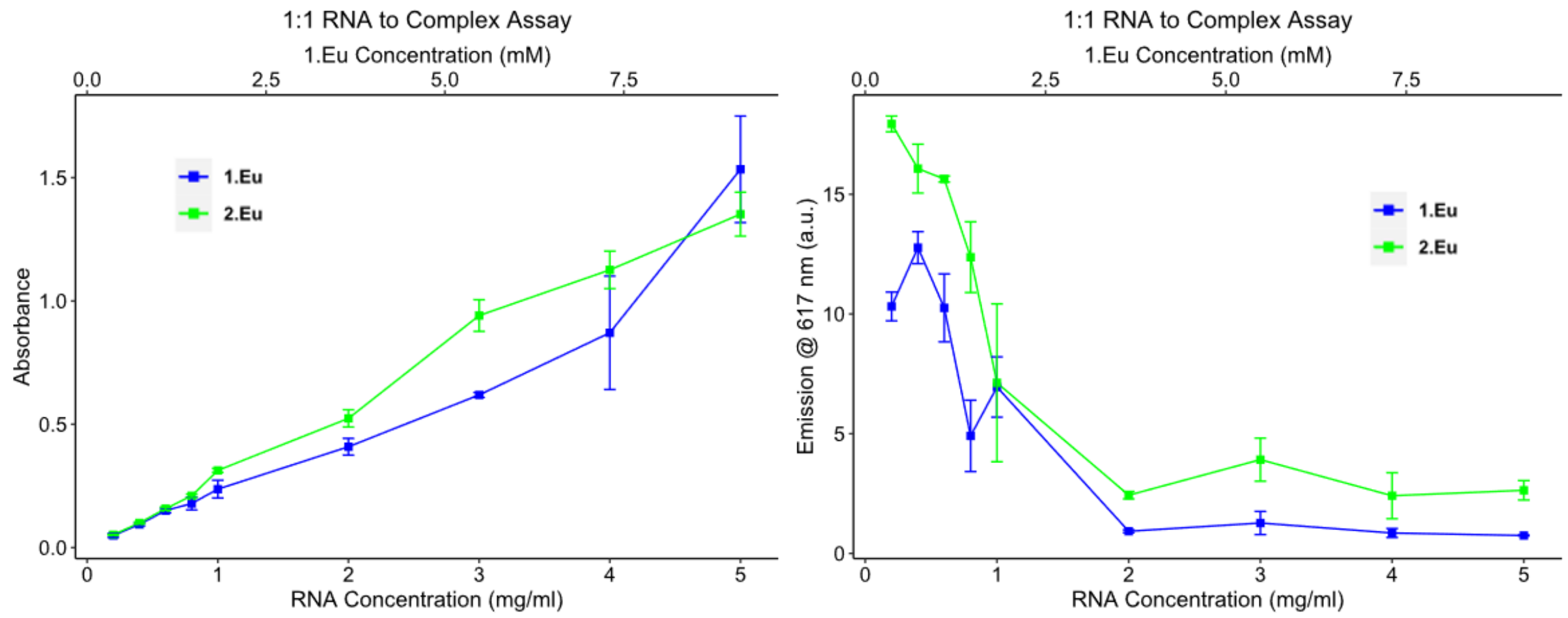

Figure S11: Absorbance at 700 nm (left) and emission (right) at 617 nm for various concentrations of **1.Eu**, **2.Eu** in aqueous HEPES (10 mM, pH 7.0)/DMSO (99:1, v/v) buffer.

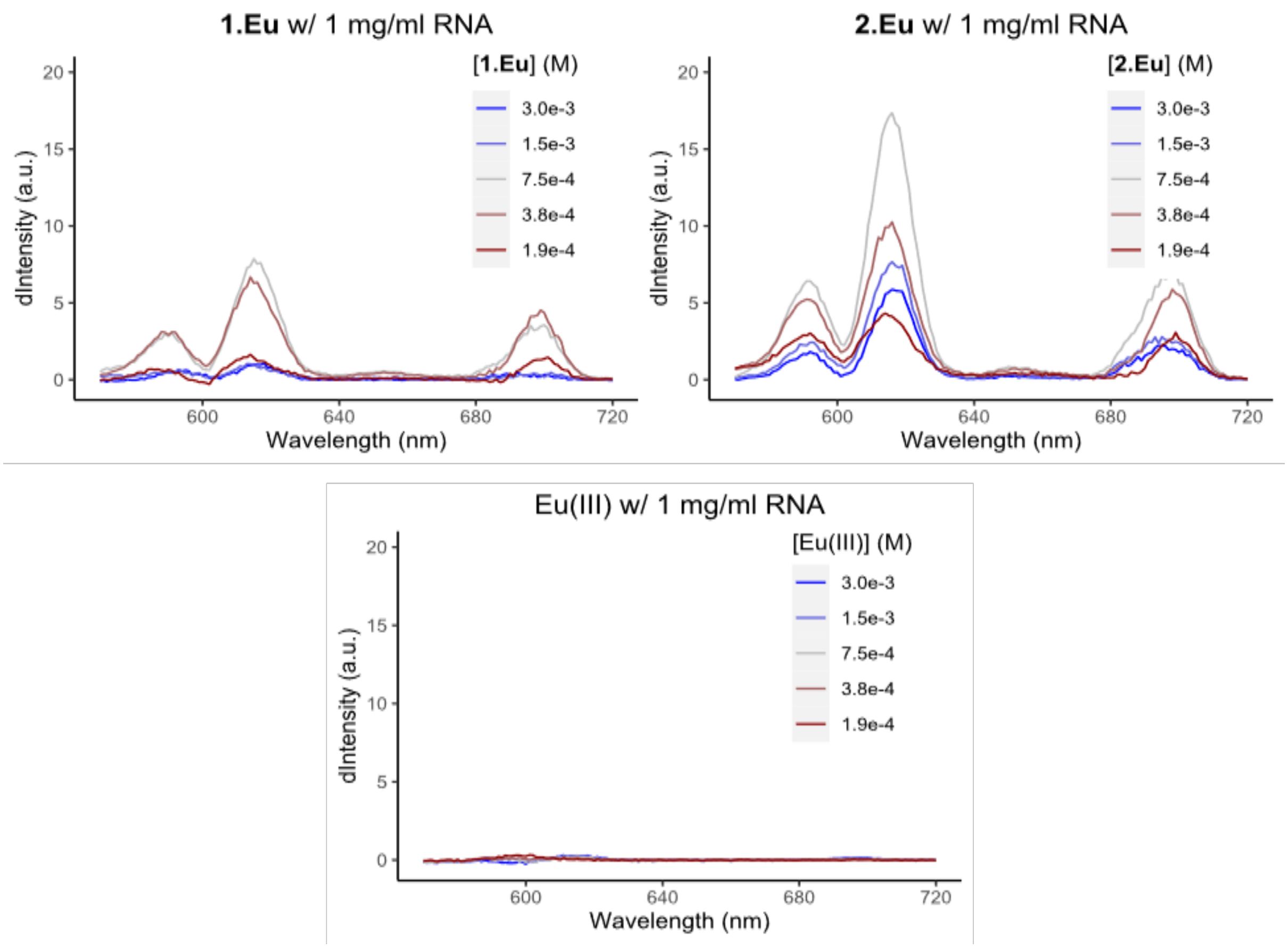

Figure S12: Emission plate reader assay for various concentrations of **1.Eu**, **2.Eu** and Eu(III) mixed with RNA in aqueous HEPES (10 mM, pH 7.0)/DMSO (99:1, v/v) buffer.

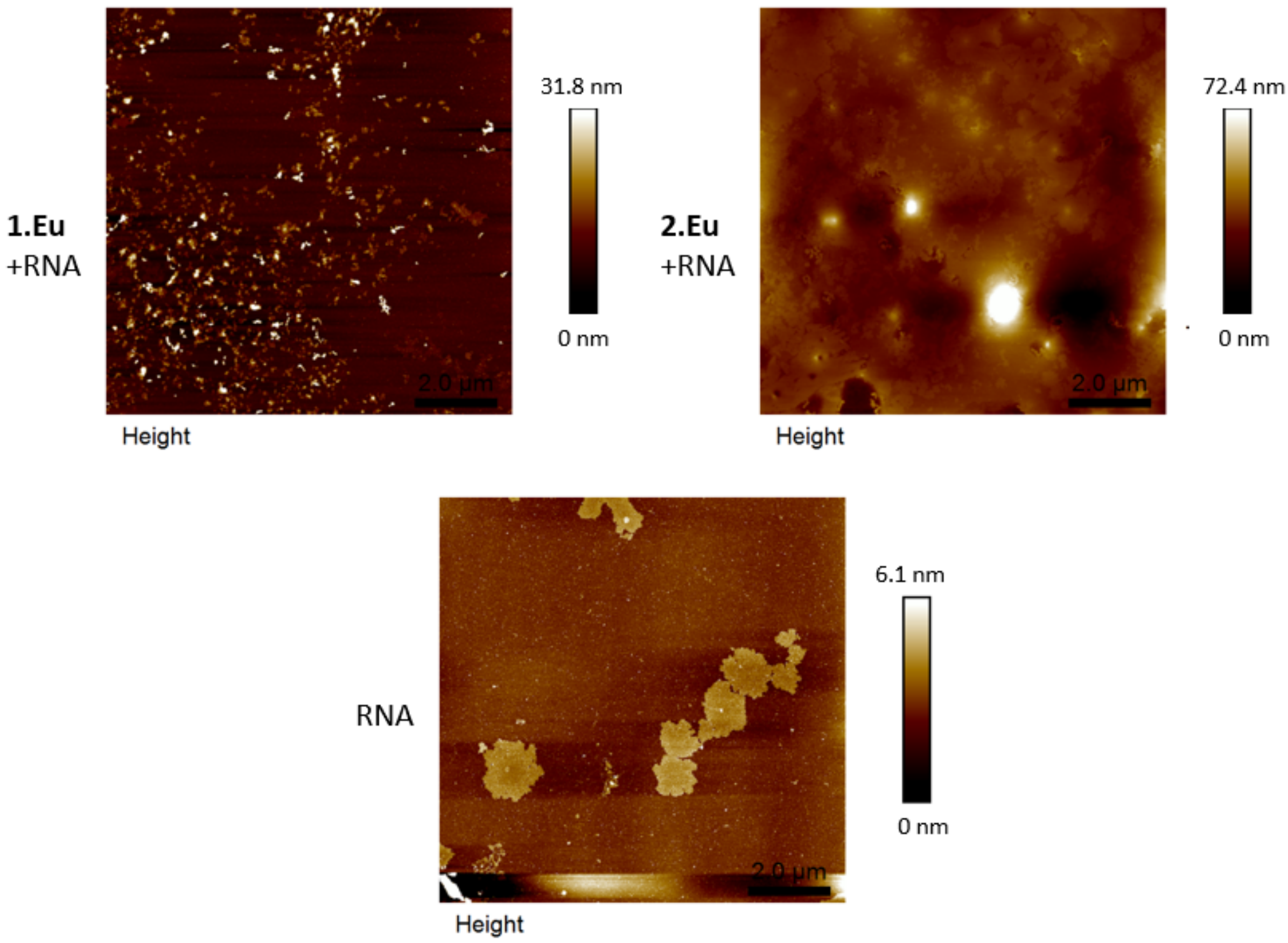

Figure S13: AFM microscopy of complex solutions **1.Eu** and **2.Eu** mixed with RNA, and the RNA control in aqueous HEPES (10 mM, pH 7.0)/DMSO (99:1, v/v) buffer.

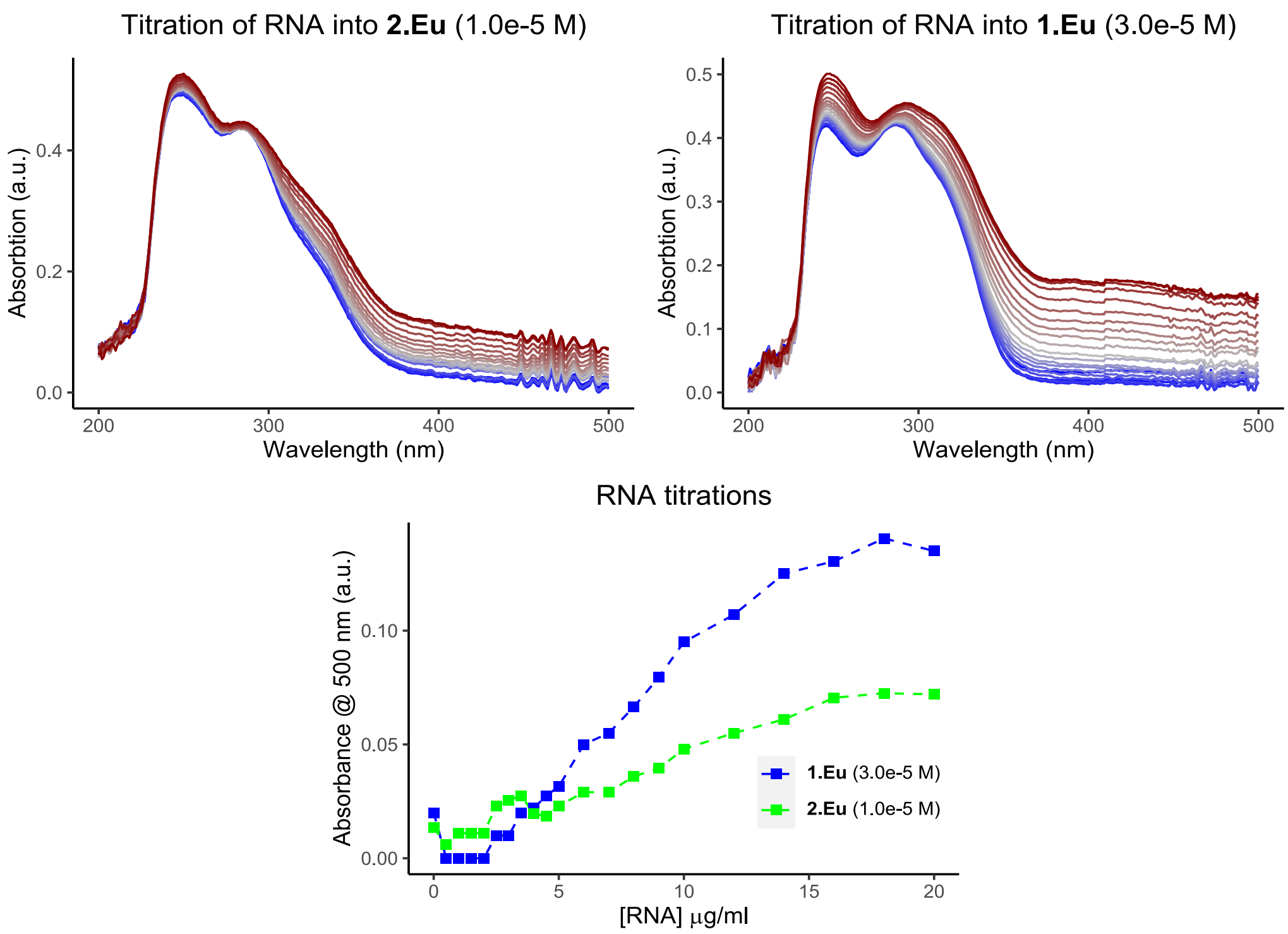

Figure S14: Absorbance measurements during the titration of RNA into solutions of **1.Eu** or **2.Eu** (top) in aqueous HEPES (10 mM, pH 7.0)/DMSO (99:1, v/v) buffer. Absorbance at 500 nm of both solutions (bottom).

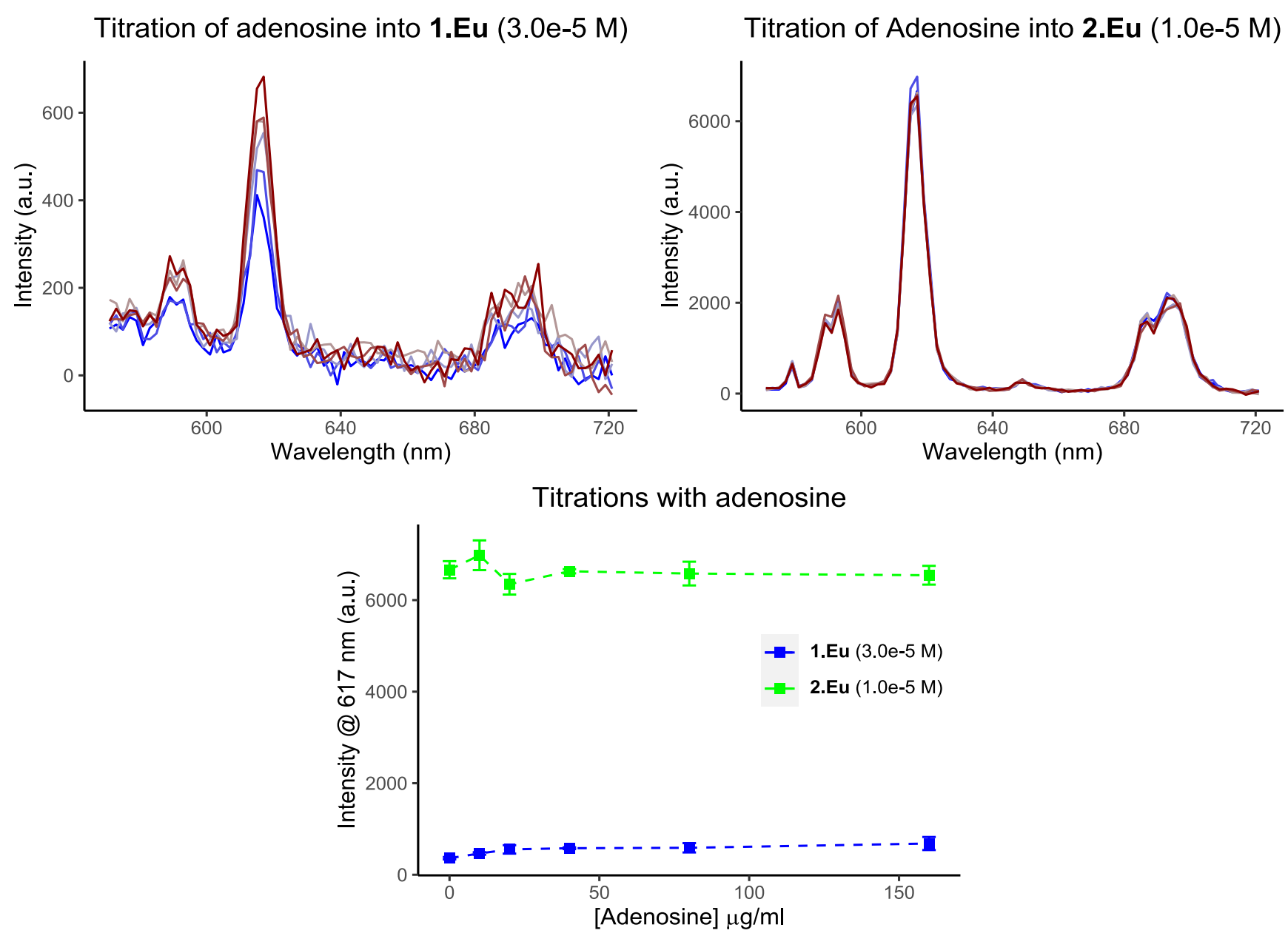

Figure S15: Emission spectra from the titration of adenosine into solutions of **1.Eu** or **2.Eu** (top) in aqueous HEPES (10 mM, pH 7.0)/DMSO (99:1, v/v) buffer. Emission tracked at 617 nm of both solutions (bottom).

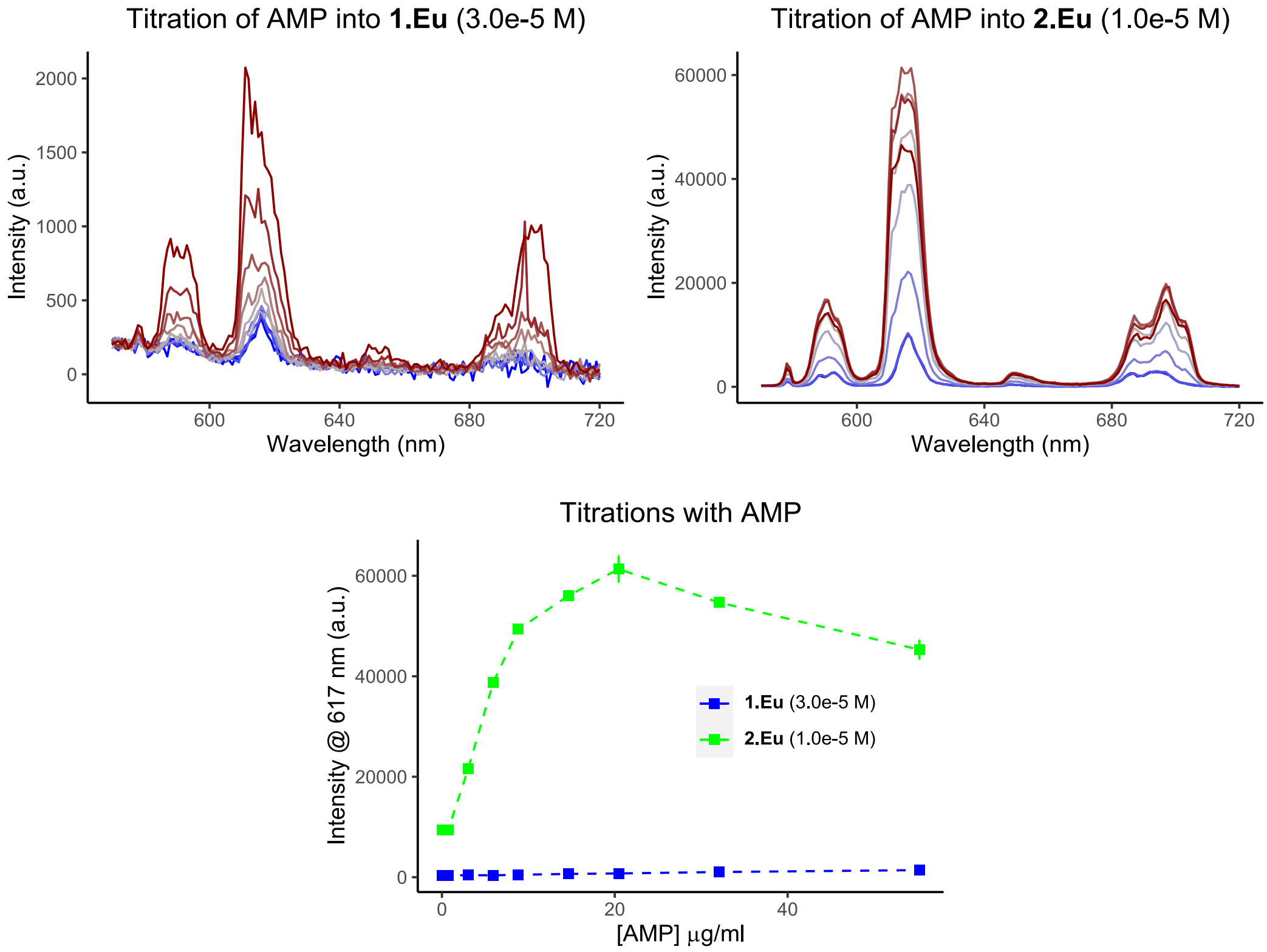

Figure S16: Emission spectra from the titration of adenosine mono-phosphate (AMP) into solutions of **1.Eu** or **2.Eu** (top) in aqueous HEPES (10 mM, pH 7.0)/DMSO (99:1, v/v) buffer. Emission tracked at 617 nm of both solutions (bottom).

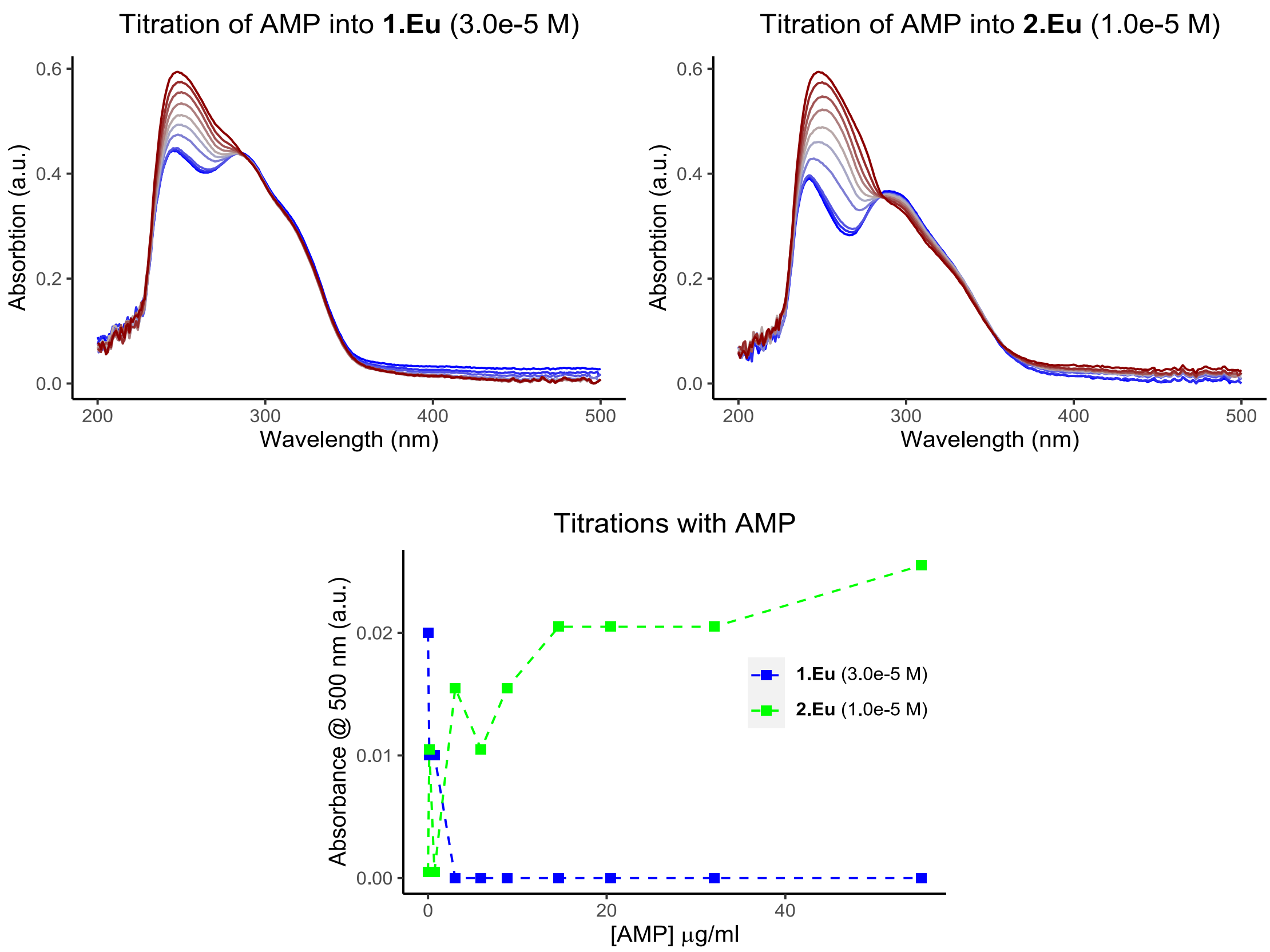

Figure S17: Absorbance spectra from the titration of adenosine mono-phosphate (AMP) into solutions of **1.Eu** or **2.Eu** (top) in aqueous HEPES (10 mM, pH 7.0)/DMSO (99:1, v/v) buffer. Absorbance tracked at 500 nm of both solutions (bottom).

Equations

$$q = 1.1 * \left[\frac{1}{\tau_{H2O}} - \frac{1}{\tau_{D2O}} - 0.31 + 0.45 * n_{OH} + 0.99 * n_{NH} + 0.075 * n_{CONH}\right] \qquad (\mathbf{S1})$$

Where q denotes the net hydration number of the complex, τ_{H2O} the emission lifetime in H_2O, τ_{D2O} the emission lifetime in D_2O, n_{OH} the number of alcohol OH oscillators in the coordination sphere, n_{NH} the number of amine NH oscillators in the coordination sphere, and n_{CONH} the number of amide CONH oscillators in the coordination sphere.

$$\theta = \frac{x^n}{K_d - x^n} \qquad K_a = K_d^{-1} \qquad (\mathbf{S2})$$

Where θ denotes the proportion of bound guest (RNA), x is the concentration of the host (the complex), n is the Hill coefficient, K_d the dissociation constant and K_a the binding constant.

$$LOD = \frac{3\sigma_0}{m} \qquad (\mathbf{S3})$$

Where LOD is the limit of detection, σ_0 is the standard deviation of 10 measurements of the background (complex without RNA), and m is the slope of the calibration line.

References

1. A. L. Moller, I. A. Middleton, G. E. Maynard, L. B. Cox, A. Wang, H. L. Li and P. Thordarson, *Biomacromolecules*, 2025, **26**, 470-479.
2. O. Kotova, R. Daly, C. M. G. dos Santos, P. E. Kruger, J. J. Boland and T. Gunnlaugsson, *Inorganic Chemistry*, 2015, **54**, 7735-7741.
3. O. Kotova, R. Daly, C. M. G. dos Santos, M. Boese, P. E. Kruger, J. J. Boland and T. Gunnlaugsson, *Angewandte Chemie International Edition*, 2012, **51**, 7208-7212.
4. D. Muller, I. Zeltser, G. Bitan and C. Gilon, *The Journal of Organic Chemistry*, 1997, **62**, 411-416.
5. Y. Tabata, S. Mitani and S. Kimura, *Journal of Peptide Science*, 2016, **22**, 391-396.
6. E. J. F. Prodhomme, A. L. Tutt, M. J. Glennie and T. D. H. Bugg, *Bioconjugate Chemistry*, 2003, **14**, 1148-1155.
7. M. P. Castaldi, S. E. Gibson, M. Rudd and A. J. P. White, *Chemistry – A European Journal*, 2006, **12**, 138-148.
